# Oligoclonal expansion of atypical Vδ2^−^ γδ T cells in Good’s Syndrome

**DOI:** 10.1038/s41467-026-74273-9

**Published:** 2026-06-11

**Authors:** Esther Bandala-Sanchez, Laura Scolamiero, Josh Chatelier, Kerry A. Ramsay, Alison Morey, Sylvia Tsang, Maureen Forde, Julian J. Bosco, Marsus Pumar, Silvia Sanchez-Ramon, Kissy Guevara-Hoyer, Jesús Fuentes-Antrás, Jack Godsell, Kymble Spriggs, Anouk von Borstel, Samantha Chan, Lauren J. Howson

**Affiliations:** 1 Walter & Eliza Hall Institute of Medical Research, Melbourne, VIC Australia; 2 Department of Medical Biology, The University of Melbourne, Melbourne, VIC Australia; 3 Department of Clinical Immunology & Allergy, Royal Melbourne Hospital, Melbourne, VIC Australia; 4 Department of Medicine, The University of Melbourne, Melbourne, VIC Australia; 5 Allergy, Asthma and Clinical Immunology Service, Alfred Health, Melbourne, VIC Australia; 6 Department of Microbiology & Immunology, The University of Melbourne, at The Peter Doherty Institute of Infection and Immunity, Melbourne, VIC Australia; 7 Melbourne Cytometry Platform, The University of Melbourne, Melbourne, VIC Australia; 8 School of Translational Medicine, Faculty of Medicine, Nursing and Health Sciences, Monash University, Melbourne, VIC Australia; 9 Department of Allergy & Immunology, Monash Health, Melbourne, VIC Australia; 10 Department of Clinical Immunology, Instituto de Medicina de Laboratorio and Health Research Institute of the Hospital Clínico San Carlos, Madrid, Spain; 11 Universidad Europea de Madrid, Department of Biosciences, Faculty of Biomedical and Health Sciences, Madrid, Spain; 12 NEXT Oncology, Hospital Universitario Quirónsalud Madrid, Madrid, Spain; 13 Division of Medical Oncology & Hematology, Princess Margaret Cancer Centre, Toronto, ON Canada; 14 Department of Infectious Diseases & Immunology, Austin Hospital, Heidelberg, VIC Australia; 15 Department of Immunology, Leiden University Medical Center, Leiden, The Netherlands

**Keywords:** Immunological deficiency syndromes, Gammadelta T cells, Immunological deficiency syndromes, Sequencing

## Abstract

Good’s syndrome is a rare adult-onset immunodeficiency characterized by thymoma, hypogammaglobulinemia, B-cell lymphopenia, and T-cell dysfunction. Despite well-characterized defects in conventional immune subsets, the impact of this disorder on unconventional T cells, including γδ T cells, remains largely unexplored. In this study, we analyse γδ T cells in 10 patients with Good’s syndrome using immunophenotyping, functional assays, and T-cell receptor (TCR)δ repertoire profiling of peripheral blood and thymoma tissue. Our analyses reveal a pronounced expansion of the Vδ2⁻ γδ T-cell compartment, composed primarily of Vδ1⁺, Vδ3⁺ and the exceptionally rare Vδ8⁺ subsets. The Vδ2⁻ cells are characterized by an activated and effector phenotype and a private and oligoclonal TCRδ repertoire. The thymoma tissue contains distinct clonotypes compared to circulation, suggesting clonal focusing in response to the tumor. Together, our findings show that γδ T-cell perturbations are integral characteristics of Good’s syndrome and broaden our understanding of immune dysregulation in this acquired immunodeficiency.

## Abstract (web summary)

Good’s syndrome is a rare adult-onset immunodeficiency, characterized by B-cell lymphopenia, T-cell dysfunction and thymomas. Here the authors examine how the severely disrupted conventional T and B cell compartments affect γδ T cells and find an expansion of the Vδ2⁻ γδ T-cell compartment composed of cells with activated and effector phenotype and a private and oligoclonal TCRδ repertoire.

## Introduction

Good’s syndrome is a rare adult-onset combined immunodeficiency characterized by the presence of thymoma, hypogammaglobulinemia, low or no B cells, an inverted CD4:CD8 T-cell ratio, and recurrent bacterial, viral and fungal infections^1^. Despite its initial description in 1954, the underlying cause and pathogenesis remain poorly defined^1^. The T-cell dysregulation has been proposed to arise from thymoma-associated disruption of thymic output, leading to altered circulating T-cell populations, as observed in other thymoma-related immune disorders^2,3^. It has also been suggested that anti-cytokine autoantibodies could contribute to T-cell dysfunction^4^. The B cell dysfunction has been attributed to defective T-cell help and a bone marrow defect arresting B cell progenitors at the Pro-B cell stage of development^5^. While no inborn errors of immunity (IEI) have been associated with Good’s syndrome, somatic mutations in key immune-related genes have been reported in certain Good’s syndrome cases^6,7^. However, the pathogenesis of Good’s syndrome remains incompletely understood, with limited investigation beyond the conventional adaptive immune cell compartments.

γδ T cells are an unconventional subset of T cells that bridge innate and adaptive immunity. In humans, they are categorized according to their T-cell receptor (TCR) δ variable (TRDV) gene usage. The predominant circulating subset comprises Vδ2⁺ γδ T cells, which typically pair with the Vγ9 chain and are considered more innate-like, as they recognize and rapidly respond to phosphoantigens in a major histocompatibility complex (MHC)-independent manner^8^. In contrast, Vδ2⁻ γδ T cells are enriched in peripheral tissues and reportedly more adaptive-like^9^, as they exhibit a more diverse TCR repertoire that can expand clonally, and recognize a broader range of antigens, most of which remain unidentified^8,10^. Together, the γδ T-cell population plays key roles in immune surveillance, tissue repair, and antitumor immunity, responding rapidly through cytokine secretion and cytotoxic granule release^8,11^.

Double-negative thymocytes give rise to both αβ and γδ T-cell lineages. γδ T cells are the first T-cell lineage detected in the thymus during embryonic development, although the exact mechanisms driving their development in humans remain poorly understood^12^. Fetal γδ thymocytes typically express public, germline-encoded TCRs with limited N-nucleotide additions and a bias toward TRDV2 usage, whereas postnatal thymocytes switch to TRDV1 usage and develop a more polyclonal repertoire^13^. In contrast, αβ T-cell development occurs later and involves tightly regulated processes of positive and negative selection that ensure MHC restriction and self-tolerance. γδ T cells, which do not undergo these same selection pressures, are instead thought to be shaped by local thymic cues that direct their effector fate^14^. Consequently, both γδ and αβ T-cell populations are likely to be affected by thymic abnormalities.

Good’s syndrome provides a natural model to explore how γδ T-cell development, phenotype, and function are shaped when key immune processes are disrupted. Despite their critical roles in host immunity and immunosurveillance, γδ T cells have been largely overlooked in Good’s syndrome. Only two studies have examined γδ T-cell proportions in the circulation: one reported normal overall γδ T-cell frequencies but an expansion of the Vδ1⁺ subset^15^, while another observed a relative increase in total γδ T cells^16^. These findings are reminiscent of γδ T-cell alterations reported in common variable immunodeficiency (CVID)^17^, where IEI cause defective B cell differentiation, but no studies have yet applied comprehensive immune profiling or repertoire-level analysis to fully characterize γδ T cells in Good’s syndrome. Given the combination of recurrent infections, thymoma, and thymic disruption in this disorder, a detailed assessment of γδ T-cell frequency, phenotype, and clonality offers a unique opportunity to understand how γδ T cells are shaped in the context of an acquired immunodeficiency that affects thymopoiesis.

To understand how Good’s syndrome affects γδ T cells, we compile a cohort of 10 Good’s syndrome patients and perform a systematic analysis of their γδ T cells in comparison to healthy donors using immunophenotyping, functional assays, and TCRδ repertoire profiling of both blood and thymoma. Our results reveal a distinct disruption of the γδ T-cell compartment in Good’s syndrome, characterized by expansion of the Vδ2⁻ subset, which comprises Vδ1^+^, Vδ3⁺ and the ultra-rare Vδ8⁺ γδ T cells. These cells exhibit an activated, functionally poised phenotype, with an oligoclonal and largely private repertoire. Thymoma tissue analysis confirmed the presence of these subsets within the tumor, although they are clonally distinct from those in circulation. Collectively, our findings uncover a unique γδ T-cell perturbation in Good’s syndrome, differing from that observed in primary immunodeficiencies, and highlight the pathological increase in Vδ2⁻ γδ T cells as a previously unrecognized hallmark of Good’s syndrome.

## Results

### High frequency of activated circulating γδ T cells in Good’s syndrome

**Fig. 1:**
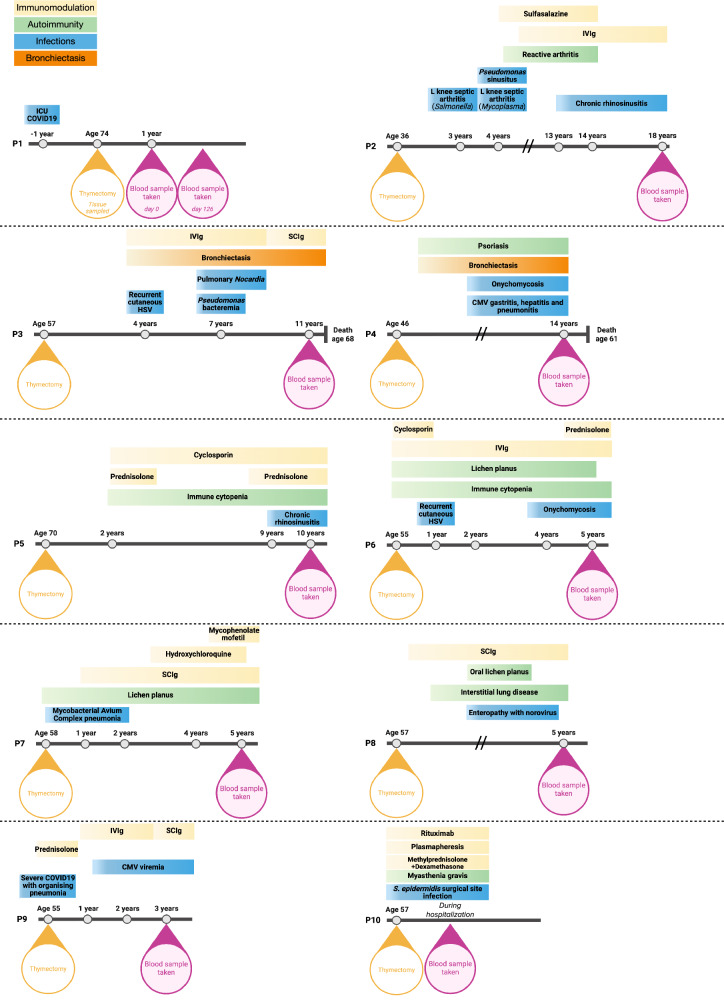
Good’s Syndrome patient clinical timelines. For each patient, the age of thymectomy and time to blood collection is outlined. Clinical infectious, inflammatory and autoimmune manifestations are provided along with administration of immunomodulating treatments. HSV, herpes simplex virus; ICU, intensive care unit; IVIg, intravenous immunoglobulin, L, left; SCIg, subcutaneous immunoglobulin. Elements in Fig. 1 were created in BioRender. Howson, L. (2026) https://BioRender.com/1ux3x89.

**Fig. 2:**
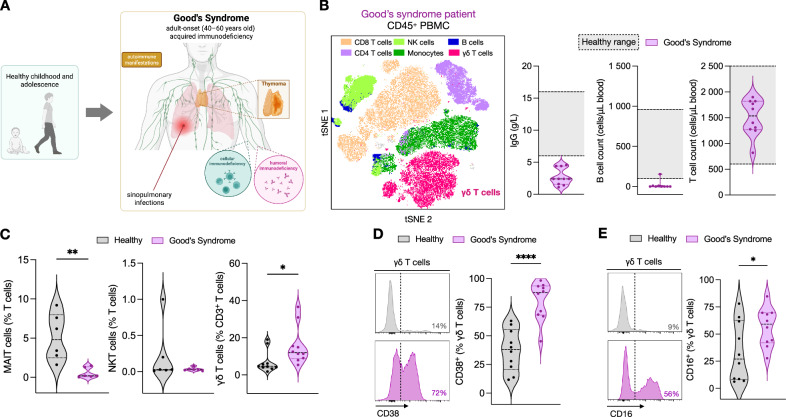
Overview of Good’s Syndrome immune cell composition. Peripheral blood mononuclear cells (PBMC) from healthy individuals (n = 6–10) and Good’s syndrome patients (*n* = 6–10) were analyzed by flow cytometry (see gating strategy in Supplementary Fig. 1A–B). **A** Overview of the clinical presentation of Good’s syndrome. **B** tSNE plot of the CD45^+^ compartment of a Good’s syndrome patient. Plots show total IgG and cell count data for Good’s syndrome patients’ B cells and T cells. **C** Frequency of circulating unconventional T-cell populations mucosal-associated invariant T (MAIT) cells, natural killer T (NKT) cells (*n* = 6 healthy, 6 Good’s syndrome) and γδ T cells (*n* = 10 healthy, 10 Good’s syndrome), where **P = 0.004 and *P = 0.034. **D** Expression of CD38 on circulating γδ T cells, where ****P = < 0.0001. **E** Expression of CD16 on circulating γδ T cells, where *P = 0.040. Statistical significance was calculated using a two-tailed unpaired T test. NK, natural killer. Panel A was created in BioRender. Howson, L. (2026) https://BioRender.com/420otiy. Source data are provided as a Source Data file.

We collected peripheral blood mononuclear cells (PBMC) from Good’s syndrome patients (see Fig. 1 for patient details and Fig. 2A for Good’s syndrome graphical summary) and healthy controls and performed immune profiling. We observed the expected severe reduction in B cells and total IgG, both hallmarks of Good’s syndrome^18^ (Fig. 2B and Supplementary Table 1), along with normal total T-cell counts but an inverted CD4:CD8 T-cell ratio, consistent with previous reports^18,19^ (Supplementary Fig. 2). We next examined the unconventional T-cell compartment to determine whether it was altered in Good’s syndrome. Mucosal-associated invariant T (MAIT) cells were significantly reduced (0.6 ± 0.3%) compared with healthy individuals (5.1 ± 1.2%), whereas natural killer (NK) T-cell frequencies were comparable between groups (Fig. 2C). In contrast, γδ T cells were significantly increased in Good’s syndrome (15.5 ± 3.2%) compared to healthy individuals (7.0 ± 1.9%) (Fig. 2C).

To characterize the phenotype of γδ T cells in Good’s syndrome we assessed their expression of CD38, a marker of activation, and the Fc receptor CD16 (FcγRIII), which mediates γδ T cells antibody-dependent cytotoxic activity. We found a higher proportion of CD38^+^ γδ T cells (81 ± 5.3%) in Good’s syndrome patients compared to healthy individuals (37 ± 5.8%) (Fig. 2D). Similarly, we observed a higher proportion of CD16^+^ γδ T cells (56 ± 5.6%) in Good’s syndrome patients compared to healthy individuals (34 ± 8.7%) (Fig. 2E). Thus, the circulating γδ T cells in Good’s syndrome patients are abundant and activated, suggesting they are poised for cytokine and cell-mediated responses.

### The Vδ2^−^ γδ T-cell subset is expanded, activated and cytotoxic in Good’s syndrome

**Fig. 3:**
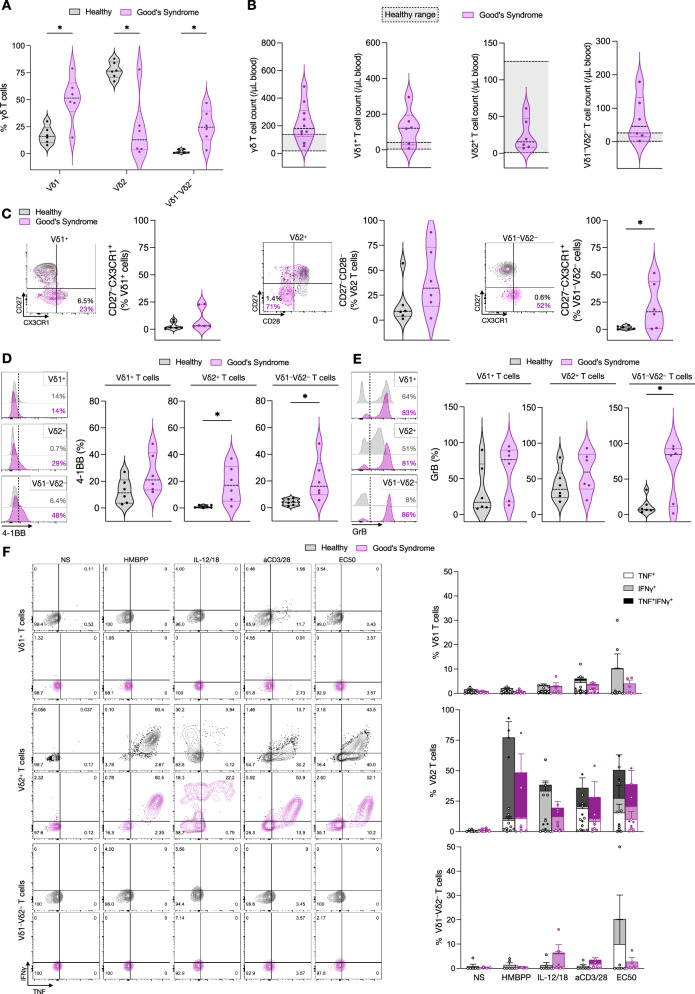
Frequency, phenotype, and function of γδ T cells in Good’s syndrome. PBMC from healthy individuals (*n* = 6) and Good’s syndrome patients (*n* = 6) were analyzed by flow cytometry. **A** Graph showing the number of Vδ1^+^, Vδ2^+^, and Vδ1^−^/Vδ2^−^ subsets as a frequency of total γδ T cells. Statistical significance was calculated using one-way ANOVA with Holm-Sidak’s multiple comparison test with single pooled variance, where *P = 0.028, 0.014 and 0.040 for Vδ1^+^, Vδ2^+^, and Vδ1^−^/Vδ2^−^ subsets, respectively. **B** Plots showing the absolute cell counts for total γδ T cells, Vδ1^+^, Vδ2^+^, and Vδ1^−^/Vδ2^−^ subsets. **C** Plots and graphs show the CD27^−^ CX3CR1^+^ Vδ1^+^, CD27^−^ CD28^−^ Vδ2^+^, and CD27^−^ CX3CR1^+^ Vδ1^−^/Vδ2^−^ effector populations, where *P = 0.028. **D** Plots and graphs show expression of 4-1BB for Vδ1^+^, Vδ2^+^ (*P = 0.013), and Vδ1^−^/Vδ2^−^ (*P = 0.026) subsets. **E** Plots and graphs show expression of granzyme B for Vδ1^+^, Vδ2^+^, and Vδ1^−^/Vδ2^−^ (*P = 0.019) subsets. **F** Plots and stacked graphs shows Vδ1^+^, Vδ2^+^, and Vδ1^−^/Vδ2^−^ subsets Th1 cytokine response to various stimuli measured by intracellular staining for TNF and IFNγ after 18 h of stimulation in the presence of brefeldin A (BFA) in healthy individuals (*n* = 5) and Good’s syndrome patients (*n* = 4). Error bars represent standard error of the mean (SEM). Statistical significance was calculated using a two-tailed unpaired T test unless otherwise stated. aCD3/28, anti-CD3/anti-CD28 dynabeads; EC50, *E. coli* multiplicity of infection 50; GrB, granzyme B; HMBPP, (E)-4-hydroxy-3-methyl-but-2-enyl pyrophosphate; IFNγ, interferon gamma; IL, interleukin; NS, no stimulation. Source data are provided as a Source Data file.

Circulating γδ T cells are predominantly innate-like Vδ2^+^ cells with a smaller adaptive-like Vδ2^−^ subset (predominantly Vδ1^+^) in healthy individuals. To determine whether the increased frequency of γδ T cells in Good’s syndrome was a true expansion driven by specific subsets, we analyzed their cell counts, frequency and phenotype. We observed a significant 2-fold increase in the proportion of Vδ1^+^ γδ T cells (51 ± 8.6%) compared to healthy individuals (17 ± 3.3%), corresponding to a significant 2-fold decrease in Vδ2^+^ γδ T cells (23 ± 12%) compared to healthy individuals (77 ± 3.1%) (Fig. 3A), the majority of which were the innate-like Vδ2^+^Vγ9^+^, similar to healthy donors (Supplementary Fig. 3). In addition, the normally minor Vδ1⁻Vδ2⁻ subset was markedly expanded in Good’s syndrome, showing an 11.5-fold increase (25 ± 6.3%) relative to healthy individuals (2.0 ± 0.7%) (Fig. 3A). When γδ T cells were assessed as absolute counts, the majority of Good’s syndrome patients exhibited elevated numbers of γδ T cells beyond that of the healthy range (Fig. 3B). This expansion was driven by increased numbers of both Vδ1⁺ and Vδ1⁻Vδ2⁻ subsets, whereas Vδ2⁺ γδ T cell counts remained within the normal healthy range across all patients. We determined that the observed increase in CD16^+^ and CD38^+^ γδ T cells was driven by the Vδ1^+^ subset (Supplementary Fig. 4).

We next investigated the proportion of effector-like γδ T-cell subsets in Good’s syndrome. The highly abundant Vδ1^−^Vδ2^−^ subset was the only population with a significantly higher proportion of effector-like (CD27^−^CX3CR1^+^) cells in Good’s syndrome (37 ± 12%) compared to healthy individuals (4.9 ± 2.4%) (Fig. 3C). The costimulatory receptor 4-1BB, which is rapidly induced on γδ T cells following antigenic stimulation and enhances their proliferation and cytokine production^20^, was also significantly upregulated in the Vδ1⁻Vδ2⁻ subset in Good’s syndrome patients (21 ± 6.3%) relative to healthy individuals (4.2 ± 1.0%) (Fig. 3D). Furthermore, the Vδ1^−^Vδ2^−^ subset had a significantly higher expression of Granzyme B at rest in Good’s syndrome (62 ± 17%) compared to healthy individuals (12 ± 4.8%) (Fig. 3E). These findings suggest that the Vδ1^−^Vδ2^−^ γδ T-cell subset in Good’s syndrome is activated and poised to participate in host immune responses using their cytotoxic potential.

We then examined the functionality of the γδ T-cell subsets by culturing them with a range of stimulants. There were no significant differences in Th1 cytokine (IFNγ and TNF) responses between Good’s syndrome patients and healthy individuals across any of the γδ T-cell subsets (Fig. 3F). As previously reported^17,21^, the innate-like Vδ2⁺ subset remained the most responsive, displaying robust activation in response to both TCR- and cytokine-dependent stimuli. In contrast, the Vδ1⁺ and Vδ1⁻Vδ2⁻ subsets showed more modest and heterogeneous cytokine responses, consistent with their adaptive-like characteristics and diverse antigen specificities^10^.

### TCRδ repertoire analysis reveals the Vδ2^−^ γδ T-cell compartment in Good’s syndrome is primarily composed of Vδ1^+^, Vδ3^+^ and Vδ8^+^ cells

**Fig. 4:**
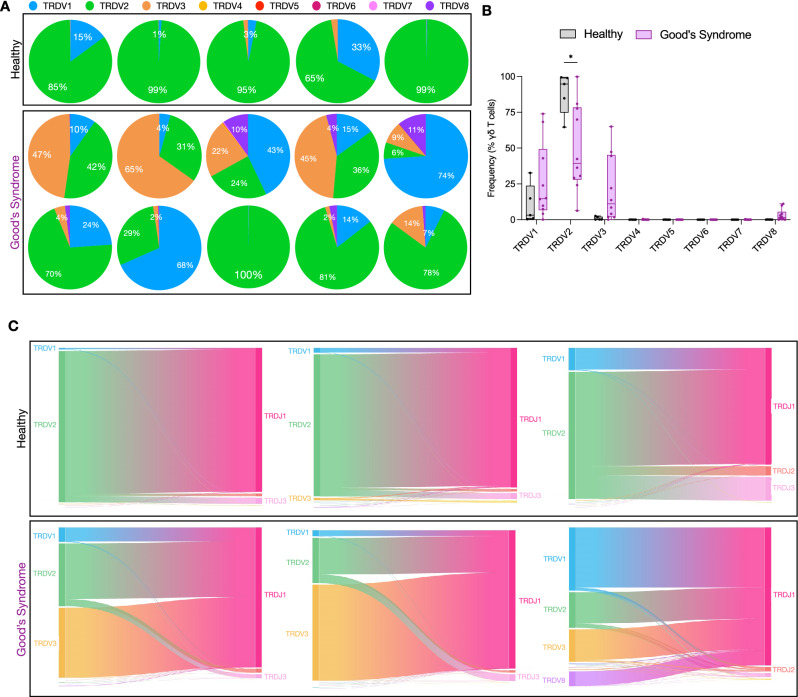
TCRδ usage by γδ T cells in Good’s syndrome. **A** TCRδ pie charts shown for γδ T cells sorted from healthy individuals (*n* = 5) and Good’s Syndrome patients (*n* = 10). **B** Graph showing the TCRδ usage comparing healthy (*n* = 5) and Good’s Syndrome (*n* = 10), where line is at median, box is upper and lower quartiles, and error bars are minimum and maximum values. Statistical significance was calculated using two-way ANOVA with Sidak’s multiple comparison test with single pooled variance, where *P = 0.041. **C** Example Sankey diagrams showing the pairing of the TRDV with TRDJ segments for healthy and Good’s syndrome γδ T cells, figure created using Flourish [https://flourish.studio]. TCR, T-cell receptor. Source data are provided as a Source Data file.

We performed TCRδ repertoire analysis on total γδ T cells to examine the distribution of TCRδ chain usage in Good’s syndrome (see Supplementary Table 2 for cell sorting and sequencing counts). In healthy adults, the circulating TCRδ repertoire is dominated by TRDV2, with a smaller contribution from TRDV1^9^, which we confirmed in our healthy cohort (Fig. 4A). In contrast, Good’s syndrome patients displayed a higher diversity of TRDV chain usage, with the majority utilizing a higher frequency of TRDV2^−^ chains (Fig. 4A), consistent with our immune phenotyping results. TRDV2 usage was significantly lower in Good’s syndrome (50 ± 9.6%) compared to healthy individuals (89 ± 6.5%) whereas the TRDV1 usage was higher (26 ± 8.4%) compared to healthy (10 ± 6.2%) (Fig. 4B). Notably, the normally minor TRDV3 was markedly increased with a 20-fold higher frequency in Good’s syndrome (21 ± 7.3%) compared to healthy individuals (1 ± 0.5%). In addition, TRDV8 usage, which is normally rare to undetectable in circulation (0.08 ± 0.03%), was elevated in Good’s syndrome patients (3.2 ± 1.3%) (Fig. 4B) indicating a distinct increase of Vδ8^+^ γδ T cells within the Vδ1^−^Vδ2^−^ compartment.

To determine which variables may influence this γδ T-cell subset frequency in Good’s syndrome, we plotted their frequency against a range of baseline and clinical variables. We found a significantly higher frequency of Vδ1⁻Vδ2⁻ γδ T cells in males compared to females (Supplementary Fig. 5A). Age was positively correlated with frequency of Vδ1^+^ γδ T cells (R^2^ = 0.44, *P* = 0.04) but did not correlate with Vδ2^+^ or Vδ1⁻Vδ2⁻ subsets (Supplementary Fig. 5B). We also found that only the Vδ1⁻Vδ2⁻ γδ T-cell frequency had a significant positive correlation (R^2^ = 0.58, *P* = 0.01) with the patients’ number of years post-thymectomy, with no correlation observed for Vδ1^+^ or Vδ2^+^ subsets (Supplementary Fig. 6A). Number of infectious complications or autoimmune complications did not significantly differ with any γδ T-cell subset frequency (Supplementary Fig. 6B–C).

We next examined the TCRδ joining (TRDJ) segment usage to assess whether γδ T cells were adult-derived (TRDJ1) or fetal-derived (TRDJ2/3)^13^. TRDJ usage patterns were similar across all subsets, with TRDJ1 predominating, consistent with an adult-derived TRD repertoire (Fig. 4C). Together, these findings demonstrate that the γδ T cells in Good’s syndrome are composed of adult-derived TRDV2^−^ subsets and primarily composed of Vδ1^+^, Vδ3⁺ cells, with 6/10 patients also having a distinct Vδ8⁺ γδ T cell population.

### The Vδ1^+^,Vδ3^+^ and Vδ8^+^ γδ T-cell subsets in Good’s syndrome are oligoclonal

**Fig. 5:**
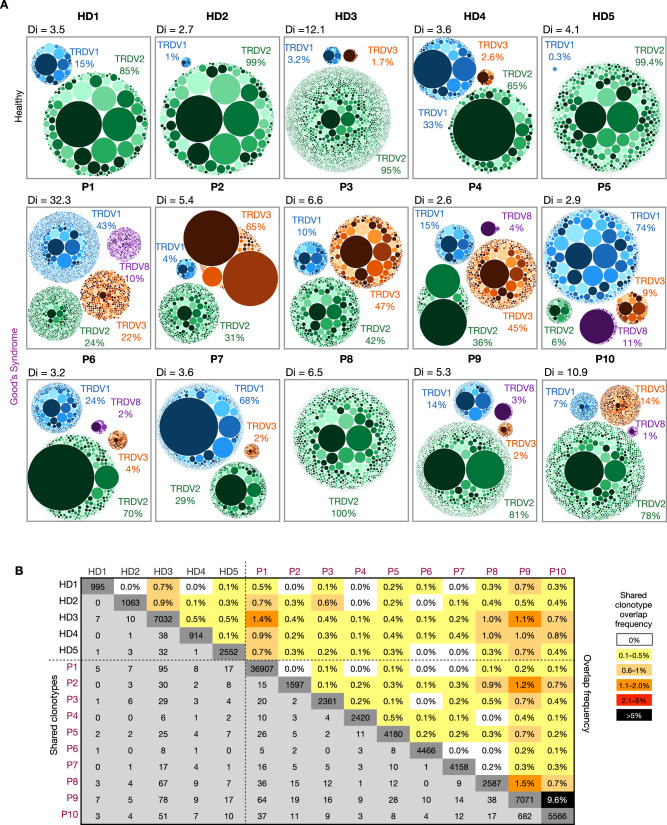
Circulating γδ T-cell repertoire in Good’s syndrome. **A** TCRδ hierarchical bubble plots shown for γδ T cells from healthy individuals (*n* = 5) and Good’s Syndrome patients (*n* = 10). Each bubble represents a unique clonotype as a proportion within the total repertoire (size of bubble). Color of clonotype bubbles group based on the TRDV usage. Figure created using Flourish [https://flourish.studio]. **B** Number and frequency of TCRδ clonotypes (where abundance >10 sequencing reads). Plot shows number of shared clonotypes (light gray), frequency of shared clonotypes (color key), and total number of clonotypes for each sample (dark gray diagonal). Di, diversity index.

We next wanted to determine whether the highly abundant γδ T-cell subsets in Good’s syndrome reflected clonal focusing of the repertoire. TCRδ clonal analysis revealed the overall diversity of the γδ T-cell repertoire was low in healthy individuals and Good’s syndrome patients, consistent with the semi-invariant and restricted nature of γδ TCR repertoires, particularly in older individuals^17^. Despite the limited diversity, most γδ T-cell subsets in Good’s syndrome were not dominated by a single clonotype but instead displayed varied clonotypes across TRDV families, including within the TRDV1 and TRDV3 subset (Fig. 5A). The top expanded clonotypes for each donor are a mixture of subsets, consisting of mostly public TRDV2 subsets and private TRDV1 and TRDV3 subsets (Supplementary Table 5).

The rare TRDV8 subset showed a dominant clonotype in patients P4, P5 (as the top expanded clonotype), and P9 but was polyclonal in P1, P6, and P10 (Fig. 5A). These findings suggest that γδ T-cell expansion and skewing in Good’s syndrome is not solely driven by clonal focusing but may also reflect altered thymic output or responses to diverse antigenic stimuli, resulting in oligoclonal expansion of atypical γδ T-cell subsets.

### The TCRδ repertoires of Good’s syndrome patients are predominantly private

To assess inter-individual variation, we compared TCRδ clonotype overlap among healthy donors and Good’s syndrome patients. Clonotype sharing was minimal across individuals (0.4 ± 1.0%) irrespective of disease status (Fig. 5B). There was an unusually high clonotype overlap between P9 and P10 of 9.6%. These were predominantly TRDV2 clonotypes with shorter CDR3 sequences that were clonally expanded in the donors (see Supplementary Table 4 for the top shared clonotype details). Overall, the γδ T-cell repertoires of Good’s syndrome patients remain largely private, indicating no evidence of disease-associated convergence or shared clonal expansions.

### γδ T cells in thymoma tissue mirror circulating subset distribution but are clonally distinct

**Fig. 6:**
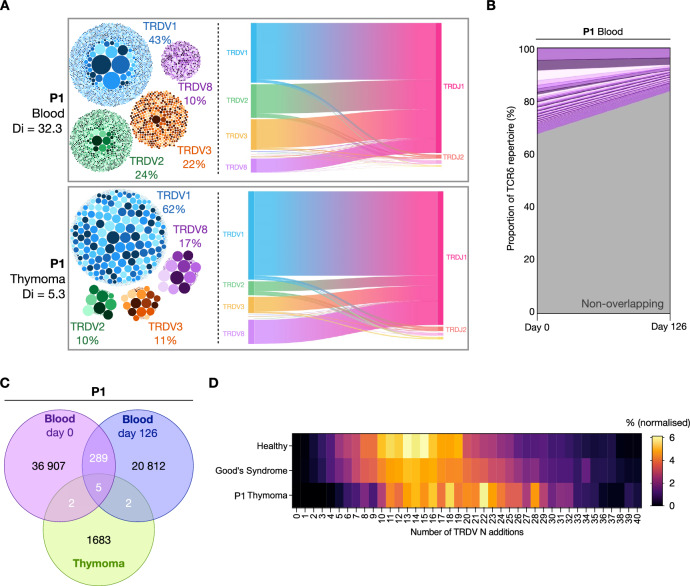
Overlap of γδ T-cell repertoire over time and with thymoma. **A** TCRδ hierarchical bubble plots shown for Good’s Syndrome patient P1 blood and thymoma (resected 1 year prior). Each bubble represents a unique clonotype as a proportion within the total repertoire (size of bubble). Color of clonotype bubbles group based on the TRDV usage. Sankey diagrams show the pairing of the TRDV with TRDJ segments. Figure created using Flourish [https://flourish.studio]. **B** Stacked bar graph shows clonotypes tracked in the blood of P1 126 days apart and compared to resected thymoma tissue. Each colored segment represents a shared clonotype, non-overlapping clonotypes are grayed out. **C** Venn diagram showing the clonotype overlap between blood (two time points) and thymoma of P1. **D** Heatmap showing the % normalized number of TRDV N additions in healthy circulating γδ T cells (*n* = 5), Good’s syndrome circulating γδ T cells (*n* = 10), and thymoma from P1. Source data are provided as a Source Data file.

For patient P1, we had access to paraffin-embedded, formalin-fixed thymoma tissue and two post-thymectomy blood draws taken 1 year after thymectomy (and 126 days apart). This enabled comparison of the γδ T-cell repertoire between tumor and blood, as well as assessment of the stability of the circulating repertoire over time. TRDV and TRDJ chain usage was broadly similar between blood and thymoma; however, thymoma-derived γδ T cells displayed lower repertoire diversity (Di = 5.3) compared with the high diversity observed in P1’s blood (Di = 32.3) (Fig. 6A). Notably, the frequency of TRDV8 γδ T cells was even higher in the thymoma (17%) compared to blood (10%) (Fig. 6A).

Our Good’s syndrome cohort comprised a range of thymoma histological subtypes and stages (Supplementary Table 2). We therefore examined whether these clinical variables were associated with alterations in γδ T cell subset distribution. Overall, we observed no significant differences according to tumor stage or histological type. The only exception was the more aggressive Stage B2 thymomas, which had significantly reduced TRDV2 usage compared with healthy controls (Supplementary Fig. 7).

Longitudinal tracking of circulating γδ T cells revealed that 20% of the repertoire was shared between the two timepoints, with dominant expanded clonotypes persisting over 126 days (Fig. 6B). In contrast, thymoma clonotypes showed minimal overlap with circulating cells from either blood draw timepoint, suggesting either expansion of tumor-specific populations in the thymoma, or an altered thymic output of γδ T cells that seed into peripheral tissues upon thymic egress (Fig. 6C).

We also compared TRDV N-addition distributions across healthy donors, Good’s syndrome patients, and P1 thymoma to assess potential VDJ recombination disruptions. Circulating γδ T cells from healthy and Good’s syndrome individuals exhibited similar N-addition patterns, consistent with a largely non-germline encoded adult repertoire (Fig. 6D). In contrast, thymoma-derived γδ T cells showed a higher proportion of N-additions, potentially reflecting oligoclonal expansion of rare or tissue/tumor-specific clonotypes (Fig. 6D).

### The transcriptional profiles of γδ T cell subsets in Good’s syndrome are largely preserved with evidence of functional modulation

**Fig. 7:**
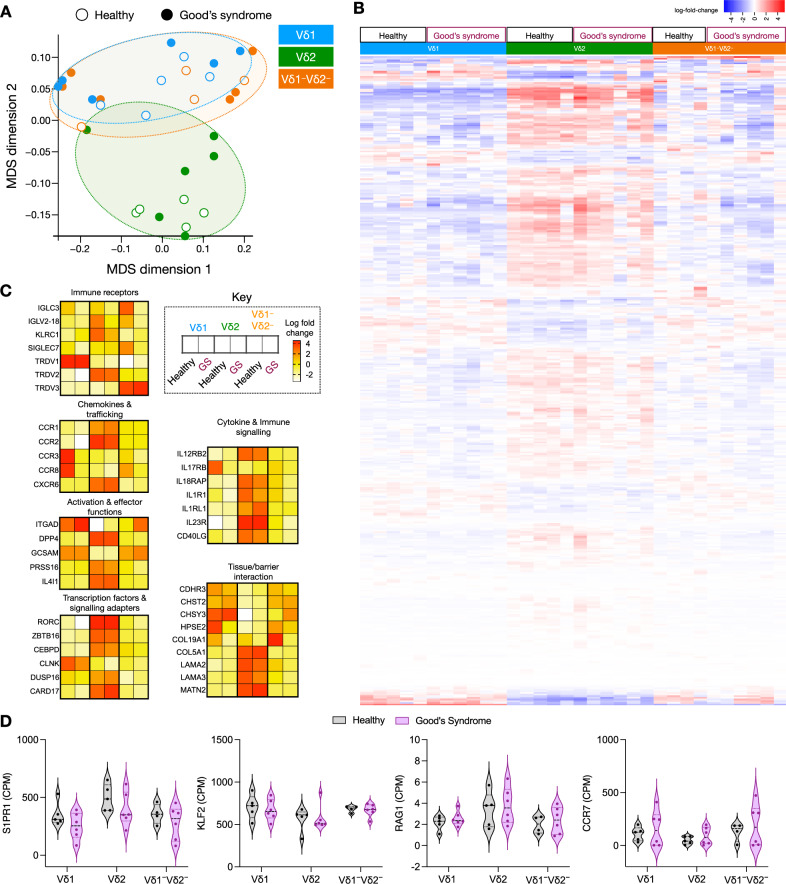
RNAseq of γδ T cell subsets in Good’s syndrome. **A** MDS plot showing samples clustering based on γδ T cell subset in healthy individuals and Good’s syndrome patients. **B** Heat map showing gene expression as log fold change relative to mean across all replicate sample γδ T cell subsets in healthy individuals and Good’s syndrome patients. **C** Heat maps showing differential gene expression ( >0.585) as log fold change relative to mean across γδ T cell subsets in healthy individuals and Good’s syndrome patients. **D** Graphs of the read counts for genes related to thymic egress: *S1PR1*, *KLF2*, *RAG1* and *CCR7*. Statistical significance was calculated using two-way ANOVA with Sidak’s multiple comparison test with single pooled variance. CPM, counts per million; GS, Goods syndrome; MDS, multidimensional scaling. Source data are provided as a Source Data file.

To determine whether the γδ T cell subsets in Good’s syndrome are functionally distinct and comparable to those observed in healthy individuals, we performed RNA sequencing on ex vivo sorted subsets. Consistent with prior reports^22^ Vδ2⁺ and Vδ2⁻ γδ T cells exhibited clear transcriptional separation, which was largely maintained between healthy individuals and Good’s syndrome patients (Fig. 7A, B). However, Vδ2⁺ cells from Good’s syndrome patients showed increased dispersion towards the Vδ2⁻ transcriptional space, suggesting partial convergence towards an adaptive-like state (Fig. 7A), in line with the expanded Vδ2⁺Vγ9⁻ population we observed in certain patients (Supplementary Fig. 3).

At the transcriptomic level, differential expression analysis revealed selective alterations in immune-related pathways. Vδ1⁺ γδ T cells from Good’s syndrome patients displayed reduced expression of *CCR3*, *CCR8*, *IL17RB* and *HPSE2* consistent with altered migratory and effector potential. In Vδ1⁻Vδ2⁻ subsets, increased *ITGAD* expression alongside reduced *IGLC3*, *SIGLEC7*, *CCR8* and *COL19A1* expression suggests shifts in adhesion, immune regulation, and tissue interaction programs. Collectively, these data indicate that while core subset-defining transcriptional programs are preserved, Good’s syndrome is associated with targeted rewiring of immune pathways, suggesting that γδ T cells retain lineage identity but undergo context-dependent functional adaptation.

We next examined the expression of genes associated with thymic egress and recent thymic emigrant status, including *S1PR1*, *KLF2*, *RAG1*, and *CCR7* (Fig. 7C). No significant differences were observed across γδ T cell subsets or between Good’s syndrome patients and healthy donors at the transcriptomic level. These findings suggest that the altered γδ T cell composition is unlikely to be driven by differences in recent thymic egress, and instead reflects prior perturbations associated with thymoma and/or peripheral adaptation following disease onset.

## Discussion

Recent studies of γδ T cells and their diverse TCR repertoires have uncovered their unique roles across infection^23–26^, cancer^27,28^, and more recently primary immunodeficiencies^17,21^. Examining γδ T cells in primary immunodeficiencies provides a unique opportunity to determine the factors that govern their development, phenotype and function in humans with immune dysfunction due to an IEI. A context in which γδ T cells have not been assessed is Good’s syndrome. As an IEI phenocopy^29^, immune function is initially normal in early to adult life, with Good’s syndrome associated dysfunction arising in later decades of life. While CVID is often considered clinically similar to Good’s syndrome due to the shared features of adult-onset hypogammaglobulinemia, it arises from intrinsic B-cell differentiation defects and occurs without thymic pathology. In contrast, Good’s syndrome combines acquired loss of B cells with thymoma-associated immune dysregulation.

Thymomas are thymic epithelial tumors categorized (A, AB, B1–3) based on the histological presentation of epithelial cells and abundance of lymphocytes. They have a low mutational burden^30^, and can sustain intratumoral hematopoiesis but they have lost immune tolerance. This is thought to drive both the autoimmune and immunodeficient manifestations of thymoma-related disorders such as myasthenia gravis and Good’s syndrome. This provides a unique model in which to examine how thymoma, disrupted central tolerance, and recurrent infections reshape γδ T-cell homeostasis and uncover new mechanisms of immune regulation in adulthood.

We observed a significant expansion of γδ T cells in Good’s syndrome patients compared to healthy individuals. This was coupled with an activated phenotype, suggesting the γδ T cells were actively participating in host immune responses. Patient clinical histories were highly variable, yet every individual displayed features of infectious and/or autoimmune complications. The increase in γδ T-cell activation is likely driven by these chronic and opportunistic immune challenges, but may also reflect a compensatory mechanism whereby γδ T cells assume a more prominent role in host immune defense in the absence of humoral immunity, similar to what has been observed in IEI^17,21^.

We found that the expansion of the γδ T cell compartment in Good’s syndrome was primarily driven by an increased frequency of Vδ2^−^ γδ T cells. This could, in part, be due to the patients’ CMV status as it is one of the most well characterized drivers of Vδ2^−^ γδ T cell clonal expansion^25^. Of the 10 patients in the Good’s syndrome cohort, 4 were determined to be CMV seropositive of the 5 tested, either by detection of CMV viraemia or serology performed in the absence of Immunoglobulin (Ig) replacement. CMV exposure could not be determined in the remaining 5 due to ongoing Ig therapy. However, in contrast to CVID with CMV viremia, where the predominant and activated subset is Vδ1^+^ cells^17^, both Vδ1^+^ and Vδ1^−^Vδ2^−^ subsets were significantly increased in Good’s syndrome. Notably, it was the Vδ1^−^Vδ2^−^ subset that exhibited an effector-like phenotype with elevated cytotoxic potential. These findings suggest that the mechanisms driving γδ T-cell subset skewing differ between CVID/CMV and Good’s syndrome. Given that both disorders feature impaired humoral immunity and recurrent infections, it is possible that the presence of the thymoma in Good’s syndrome reshapes the γδ T-cell subset composition. This may occur through altered thymic output, potentially driven by aberrant expression of autoantigens or disruption of normal thymic selection cues provided by thymic epithelial cells. Alternatively, changes in subset composition could reflect tumor-associated antigen-driven expansion, including responses to dysregulated self-antigens, stress-induced ligands, or tumor neoantigens recognized by γδ TCRs. However, as the antigenic landscape of thymomas in Good’s syndrome is poorly understood, it warrants further investigation.

Good’s syndrome patients exhibited a marked shift in TRDV gene usage, with increased TRDV1, TRDV3, and TRDV8. The oligoclonal expansions of Vδ3^+^, and particularly Vδ8^+^, was unexpected as this pattern diverges from what has been shown in other primary immunodeficiencies that reported expansion of γδ T cells^17,21^. Notably, a Vδ8^+^ γδ T-cell population has not been previously described in humans outside of γδ T cells isolated from acute myeloid leukemia patients^31,32^. Our findings therefore identify Good’s syndrome as a new pathological context in which Vδ8⁺ γδ T cells are elevated.

The increased frequency of atypical Vδ1^+^, Vδ3^+^ and Vδ8^+^ γδ T cells in Good’s syndrome patients may result from thymoma-driven disruptions to thymic selection and output. The private and clonally unfocused nature of these subsets suggest broad immune dysregulation affecting this population. While γδ T cells have not been previously studied in the context of Good’s syndrome, they have been characterized in other thymoma patients. A study by Christopoulos et al^33^. reported a thymoma patient with an increase in circulating γδ T cells (30% of total T cells), with the majority being polyclonal expansions of Vδ1^+^ and Vδ3^+^. Based on the unusual TRDV usage, naïve phenotype, and polyclonal nature, they concluded that these cells likely arose from thymoma-induced alterations in thymic output^33^. Of note, our patients’ Vδ2⁻ T cells differed from this, exhibiting a more effector-like phenotype. This difference may reflect the wide range of sampling times post-thymectomy in our cohort, which extended up to 18 years. In this time, these thymoma-altered γδ T-cell subsets could continue expanding and actively participate in host immune responses. Supporting this, we observed that the frequency of Vδ1⁻Vδ2⁻ γδ T cells increased proportionally with the number of years since thymectomy. Another potential driver of oligoclonal expansion of atypical γδ T cells that we did not assess is the presence of somatic mutations in key immune signaling genes, such as *STAT3* and *STAT5B*. Such mutations have been reported in lymphoproliferative disorders involving Vδ2⁻ γδ T cells^34^ and given the suggested role of somatic mutations in the pathogenesis of Good’s syndrome, this represents an important avenue for future investigation.

We observed similar TRDV/TRDJ usage between thymoma and blood in patient P1, but the clonotypes were mostly non-overlapping, suggesting focusing within the thymoma. Although γδ T cells are known to participate in immunosurveillance as tissue-resident populations, this has primarily been reported in non-lymphoid peripheral tissues such as liver and mucosal sites^12^. Therefore, the γδ T cells detected in the thymoma are likely either (thymic-derived) γδ thymocytes or tumor-infiltrating γδ T cells. γδ T cells have been implicated as major players in tumor immunity, capable of recognizing and killing tumor cells via TCR-dependent or NK receptor–mediated mechanisms^35^. Tumor-infiltrating γδ T cells are typically Vδ1⁺ and exhibit superior antitumor activity compared with Vδ2⁺ cells^36^. Given the lack of clonotypic overlap with circulating cells, the thymoma γδ T cells may represent minor thymic-derived populations that selectively expanded in response to the tumor. However, the conclusions that can be drawn from these data are limited, as the analysis was performed on a single thymoma sample. Moreover, because RNA was extracted from formalin-fixed paraffin-embedded tissue sections, we were unable to assess the frequency or phenotypic characteristics of γδ T cells within the broader thymoma tissue.

The γδ T cell repertoire in Good’s syndrome patient P1 was partially stable post-thymectomy, with 20% of the repertoire being detectable across 126 days. The repertoire of γδ T cells is known to be very stable over time^37^, but can be influenced by infections, which can drive either polyclonal expansion, as observed in malaria^23^ or clonal focusing, as seen with CMV^25^. P1 did not report significant infections post-thymectomy; however, because these samples were collected within one year of thymoma removal, their γδ T-cell compartment may still have been undergoing dynamic readjustments in response to this.

We found that subset-defining transcriptional signatures of γδ T cells are largely preserved in Good’s syndrome. Despite marked alterations in subset frequency compared to healthy individuals, most notably within the Vδ1⁻Vδ2⁻ compartment, the presence of thymoma and associated immunodeficiency did not fundamentally disrupt their core transcriptional identities. Together with our findings of preserved thymic egress signatures, this suggests that γδ T cell alterations in Good’s syndrome are driven by extrinsic disease-associated factors. While altered thymic output may contribute to changes in subset distribution and TCRδ usage, this does not appear to extend to intrinsic reprogramming of γδ T cells through disrupted thymic selection or developmental programming.

Good’s syndrome remains an enigmatic immunodeficiency, with its underlying cause and pathogenesis unknown. Its characteristic immune abnormalities of hypogammaglobulinemia, low B cells, and an abnormal CD4:CD8 T-cell ratio are well established. However, the role of γδ T cells has been largely overlooked, limited to a small number of studies reporting their increase in select patients^16,38^. Our study provides the first comprehensive analysis of the γδ T-cell compartment in Good’s syndrome, revealing a distinct disruption of increased frequency of atypical Vδ3⁺ and Vδ8⁺ subsets, detectable in circulation up to 18 years post-thymectomy and within thymoma tissue. The alterations in the γδ T-cell compartment are distinct from what has been reported in the clinically similar condition CVID^17^ or any other pathological condition, highlighting a previously unrecognized immune perturbation specific to Good’s syndrome. Whether these γδ T-cell changes arise in response to the thymoma itself, reflect aberrant thymic output, or somatic mutations driving their expansion, remains to be determined. Collectively, our study has identified oligoclonal expansion of atypical γδ T-cell subsets as a hallmark of Good’s syndrome, extending the spectrum of immune dysfunction beyond conventional B and T cells, and underscoring the role of γδ T cells in anti-tumor immunity and immunodeficiency.

## Methods

### Study design

Patient samples and clinical data were obtained from the Royal Melbourne Hospital, Alfred Health, Monash Health, and the Hospital Clínico San Carlos. Good’s Syndrome was defined as thymoma associated with adult-onset immunodeficiency including hypogammaglobulinemia, consistent with the original case description^39^ and current commonly accepted criteria^4,29^ (see Fig. 1 for patient clinical timelines and Table 1 for cohort characteristics). Healthy donors were recruited through the volunteer biospecimen donor registry at the Walter and Eliza Hall Institute of Medical Research (WEHI). Clinical data and sample tracking information was stored on a REDCap database (Vanderbilt University, v14.1.5).

**Table 1:** Summary of cohort characteristics

Characteristics		Good’s syndrome *n* = 10	Healthy controls *n* = 10
Age (years: median, range)		63, 53–79	42, 28–66
Sex (*n*, [percentage])	Female	4 [40%]	5 [50%]
	Male	6 [60%]	5 [50%]
Age at diagnosis of Good’s syndrome/thymoma (years: median, range)		57, 35–72	N/A
Years since diagnosis (mean, range)		7.2, 0–18	N/A

*N/A* not applicable.

Healthy donor age, sex, and medical history was self-reported and selection for inclusion in this study was based on obtaining a balance of ages and sexes across individuals with no known immunological or autoimmune conditions. Ethical approval for this study was granted by the Human Research Ethics Committees of Melbourne Health (project ID: 2009.162), WEHI (project ID: 25/26), and Hospital Clinic San Carlos (20/072-E). Written, informed consent was obtained from all participants, in accordance with the Declaration of Helsinki prior to their participation in the study. Sample sizes were limited by the availability of eligible patients, but adequate to detect statistically significant differences between sample groups. Healthy donor ranges for cell counts are derived from clinical guidelines or in the case of γδ T cell subsets, from published healthy cohort data^40^.

### Human sample processing

Blood samples were collected via venipuncture and PBMCs were isolated by density gradient centrifugation using Ficoll-Paque Plus (Cytiva) and cryopreserved in liquid nitrogen.

### CMV serotyping

Plasma samples from healthy individuals were thawed and serotyped using an anti-CMV IgG human enzyme-linked immunosorbent assay (ELISA) kit (ab108724, Abcam) following manufacturer’s instructions. Samples were considered seropositive when absorbance values were 10% above the cut-off control.

### Antibodies and staining reagents

All antibodies used were commercially available and validated for specificity by the manufacturer. BD OptiBuild™ Brilliant Ultraviolet (BUV)615 anti-human CD27 (O323, 1:50), BD OptiBuild™ BUV805 anti-Human CD161 (DX12, 1:25), BD Horizon™ Brilliant Violet (BV)786 anti-human IFN-γ (4S.B3, 1:20), BD Horizon™ BV786 anti-human CD38 (HIT2, 1:25), and BD OptiBuild™ RealBlue (RB)613 anti-Human CD137 [4-1BB] (4B4-1, 1:25) were purchased from BD Biosciences. Alexa Fluor 700 anti-human CD28 (CD28.2, 1:25), allophycocyanin (APC) anti-human/mouse granzyme B (QA16A02, 1:10), BUV395 anti-human CD3 (SK7, 1:50), BV605 anti-human TCR Vα7.2 (3C10, 1:25), BV650 anti-human CX3CR1 (2A9-1, 1:25), PE/Dazzle 594 anti-human TNF-α (MAb11, 1:20), PE/Cyanine5 anti-human TCR Vγ9 (B3, 1:10) PE/Cyanine7 anti-human CD4 (SK3, 1:25), PerCP/Cyanine5.5 anti-human CD8 (SK1, 1:25) and PerCP/Cyanine5.5 anti-human CD16 (3G8, 1:25) were purchased from BioLegend. APC-Vio770 anti-human TCR Vδ1 (REA173, 1:10), Vioblue TCR Vδ2 (123R3, 1:100), and fluorescein isothiocyanate (FITC) anti-human TCRγ/δ (REA591, 1:10) were purchased from Miltenyi Biotec. PBS-57–loaded CD1d tetramers and 5-OP-RU–loaded MR1 tetramers were obtained from the NIH Tetramer Core Facility. Dead cells were excluded using the viability dye Zombie Aqua™ Fixable Viability Kit (BioLegend) according to the manufacturer’s instructions. Immunoprofiling was performed using antibodies from the 25-Color Immunoprofiling Assay, cFluor® Reagent Kit (Cytek) and dead cells excluded using the ViaDye™ Red Fixable Viability Dye Kit.

### Flow cytometry and cell sorting

For flow cytometry, cells were thawed and stained with viability dye for 10 min at room temperature, followed by antibodies diluted in phosphate buffered saline (PBS) with 10% fetal bovine serum (FBS) (Sigma) for 20 min on ice. Cells were fixed and permeabilized using the eBioscience™ Foxp3 / Transcription Factor Staining Buffer Set (Invitrogen) according to manufacturer’s instructions. Intracellular antibodies were then incubated for 45 min at room temperature diluted in permeabilization buffer. Flow cytometry data was collected on a Cytek Aurora running SpectroFlo software (Cytek). For the 25-Color Immunoprofiling Assay, cFluor® Reagent Kit (Cytek), cells were thawed and stained according to manufacturer’s instructions and data acquired during cell sorting on a Cytek Aurora™ CS System. All data analysis was performed using FlowJo™ software (BD, v10). Cell sorting for the RNAseq experiment was performed on a BD FACSAria Fusion.

### Bacterial culture

*Escherichia coli* was grown (at 37 °C in Lysogeny broth (LB) in shaking incubator) overnight, then diluted 1:100 in LB and grown to log phase for 3 h. OD_600_ was measured and used to determine concentration.

### γδ T-cell activation assay

PBMCs were cultured in RPMI 1640 medium (Gibco) supplemented with 10% FBS and 100 U/mL penicillin/streptomycin (Gibco) (at 37 °C and 5% CO_2_ in a humidified incubator) and pulsed with a range of stimulants. For cytokine stimulation: 100 U/mL human IL-2 (Abcam), 200 ng/mL human IL-12 (Miltenyi Biotec) and 200 ng/mL human IL-18 (BioLegend) were pulsed in two doses and cultured 72 h apart, and brefeldin A (BFA) (BioLegend) added 1 h after the second dose, before culturing for 18 h. For bacterial stimulation: *E. coli* at multiplicity of infection (MOI) 50 was added to PBMCs, cultured for 1 h prior to adding 50 μg/mL gentamicin (Sigma) and BFA and cultured for 18 h. For antigen stimulation: 10 ng/mL of HMBPP (Cayman Chemicals) was added and cultured for 1 h before adding BFA and cultured for 18 h. For TCR stimulation: Human T-Activator CD3/CD28 Dynabeads (Gibco) were cultured at a 1:1 ratio with cells for 1 h prior to addition of BFA and cultured for 18 h. Following stimulation, cells were collected and stained for flow cytometry.

### TCR repertoire analysis

γδ T cells were bulk-sorted into RNA*later* (Sigma-Aldrich) (See Supplementary Fig. 1A for gating strategy). RNA was extracted using an RNAmicro kit (Qiagen) according to the manufacturer’s instructions. RNA was extracted from formalin-fixed, paraffin-embedded thymoma tissue sections (scraped from glass slides) using the RNeasy FFPE Kit (Qiagen) according to the manufacturer’s protocol.

The human TCRδ chain iR profile kit (iRepertoire Inc.) was used to perform amplicon rescued multiplex–PCR to generate CDR3 amplicon libraries for sequencing following the manufacturer’s instructions. Sequencing was performed using an Illumina MiSeq system. Raw sequencing data files were analyzed using iRweb (iRepertoire Inc.) to assign CDR3 sequences, variable (V), diversity (D), and junction (J) gene usage, diversity index (Di) [= 100 − area under the curve between % total reads and % unique CDR3s], and tabulate clonotypes for tracking. Flourish Software (Canva UK Operations Ltd) was used to generate bubble plots and Sankey diagrams.

### RNA-seq

γδ T cell subsets were bulk-sorted into RNA*later* (Sigma-Aldrich) (See Supplementary Fig. 1B for gating strategy). RNA was extracted using an RNAmicro kit (Qiagen) according to the manufacturer’s instructions. Samples were submitted to AGRF for RNA quality check on an Agilent Bioanalyzer 2100, library preparation using Illumina Stranded Total RNA prep with Ribo-Zero Plus. Sequencing was performed using an Illumina NovaSeq X Plus with 150 bp paired end read.

### RNA-seq analysis

Raw sequencing files were trimmed and low-quality reads filtered using Trim Galore and aligned to the Ensembl Human GRCh38 genome using STAR aligner (v2.3.5a). Consensus transcripts were assembled using StringTie and number of reads mapped using FeatureCounts. The resulting gene count matrix was uploaded to the Degust web tool^41^ using the voom/limma method for testing significant differential expression analysis. A false discovery rate (FDR) of 0.05 cut-off was implemented calling differentially expressed genes. Heatmaps show log2 fold change in gene expression relative to mean, averaged between biological replicates (*n* = 4–6).

### Statistical analysis

Statistical analysis was performed using Prism (GraphPad Software, v10). For single comparison analysis, two-tailed unpaired *T* tests were used. For multiple comparisons with one independent variable, one-way analysis of variance (ANOVA) with Holm-Sidak’s multiple comparison test was used. For multiple comparisons with two independent variables, two-way ANOVA with Geisser-Greenhouse correction and Sidak’s multiple comparison test was used. For all analysis, a significant result was indicated when *P *< 0.05.

### Reporting summary

Further information on research design is available in the Nature Portfolio Reporting Summary linked to this article.

## Supplementary information

Supplementary Information

Reporting Summary

Transparent Peer Review file

## Source data

Source Data

## Data Availability

The γδ TCR repertoire raw sequence data supporting the findings of the study have been deposited in the NCBI Sequence Read Archive (SRA) under the BioProject Accession Number: PRJNA1390857. The RNA-seq raw sequence data has been deposited in the NCBI SRA under the BioProject Accession Number: PRJNA1446171. Source data are provided with this article. Source data are provided with this paper.
