# Supplementary material for: Oligoclonal expansion of atypical Vδ2− γδ T cells in Good’s Syndrome
Source: Nat Commun. 2026 Jun 11;17:7439. doi: 10.1038/s41467-026-74273-9 (PMC13407883; doi:10.1038/s41467-026-74273-9)
Supplement: Supplementary file 1 — Supplementary Information [file 41467_2026_74273_MOESM1_ESM.pdf]

Supplementary information for

Oligoclonal expansion of atypical Vδ2^{-} γδ T cells in Good's Syndrome

Esther Bandala-Sanchez, Laura Scolamiero, Josh Chatelier, Kerry A. Ramsay, Alison Morey, Sylvia Tsang, Maureen Forde, Julian J. Bosco, Marsus Pumar, Silvia Sanchez-Ramon, Kissy Guevara-Hoyer, Jesús Fuentes-Antrás, Jack Godsell, Kymble Spriggs, Anouk von Borstel, Samantha Chan & Lauren J. Howson*

*Corresponding author, email: howson.l@wehi.edu.au

The PDF file includes:

Supplementary Figure 1 – T cell gating and sorting strategies.
Supplementary Figure 2 – Circulating cell counts in Good's syndrome.
Supplementary Figure 3 – Vγ9 frequency among Vδ2^{+} γδ T cells.
Supplementary Figure 4 – γδ T cell subset expression of CD16 and CD38.
Supplementary Figure 5 – Impact of patient age and sex on the γδ T cell subsets in Good's syndrome patients.
Supplementary Figure 6 – Impact of patient clinical variables on the γδ T cell subsets in Good's syndrome patients.
Supplementary Figure 7 – Impact of thymoma type and stage on the γδ T cell subsets in Good's syndrome patients.
Supplementary Table 1 – Cell counts for Good's syndrome patients.
Supplementary Table 2 – Good's syndrome patient clinical and demographic details.
Supplementary Table 3 – Sample details for TRD repertoire analysis.
Supplementary Table 4 – Top shared clonotypes between P9 and P10.
Supplementary Table 5 – Top expanded clonotypes for each patient.

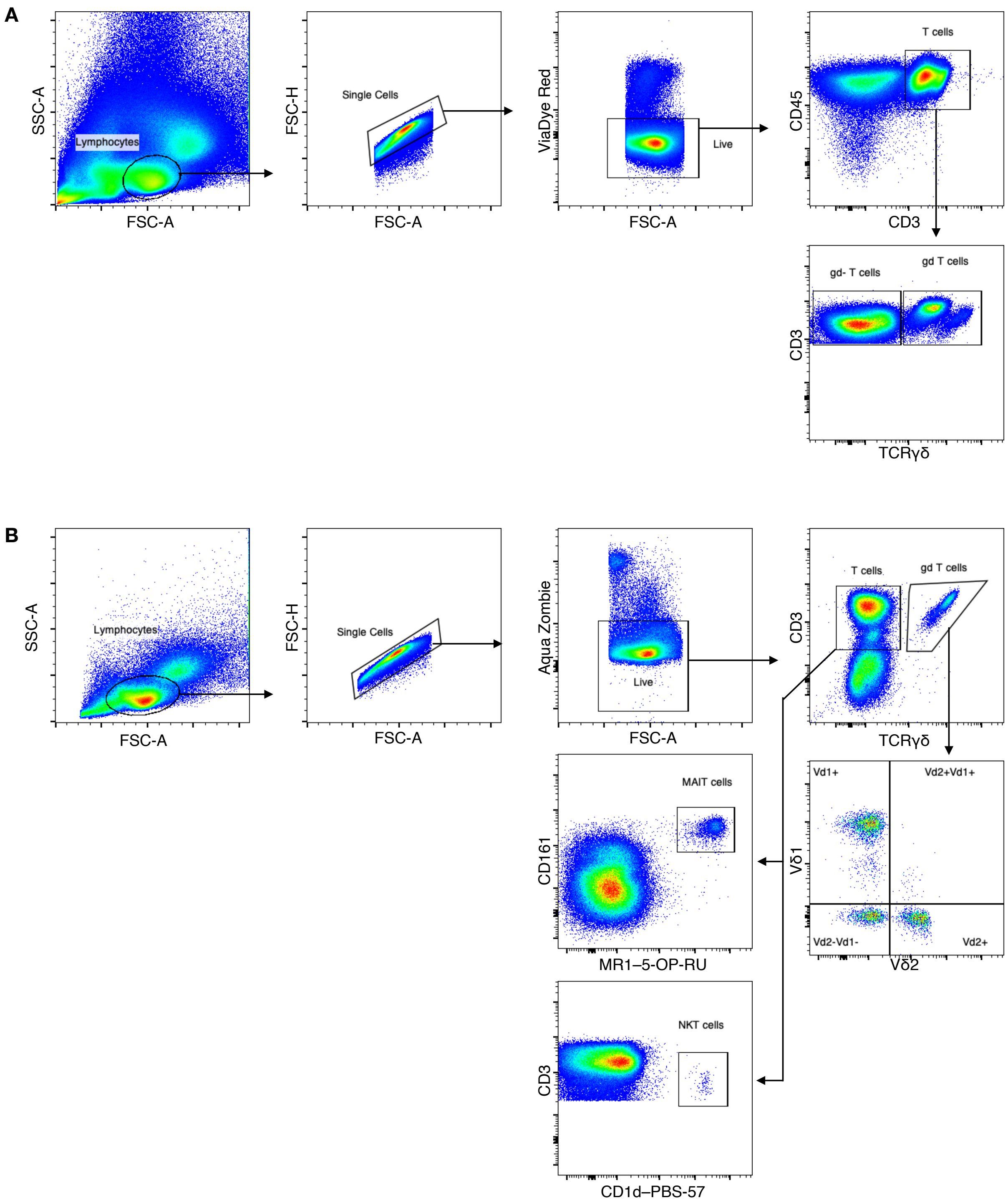

Supplementary Figure 1 – T cell gating and sorting strategies. Plots show the gating strategy of peripheral blood mononuclear cells (PBMC) samples using (**A**) the Cytek immunoprofiling kit and (**B**) our unconventional T cell panel. Cells were gated based on forward and side scatter, single cells, live, and CD3^{+} T cells and then for unconventional T cells, gating on: MAIT cells, NKT cells and γδ T cells (with Vδ1^{+}, Vδ2^{+} and Vδ1^{-}Vδ2^{-} subsets). (A) Gating strategy was used for flow cytometry experiments in Figure 2B–D and γδ sorting strategy for TCR repertoire analysis in Figure 4–6. (B) Gating strategy was used for subset analysis in Figure 2C, 3A–F and sorting for RNAseq experiment in Figure 7. FSC-A, forward scatter (area); MAIT, mucosal-associated invariant T (cell); NKT, natural killer T (cell); TCR, T cell receptor; SSC-A, side scatter (area).

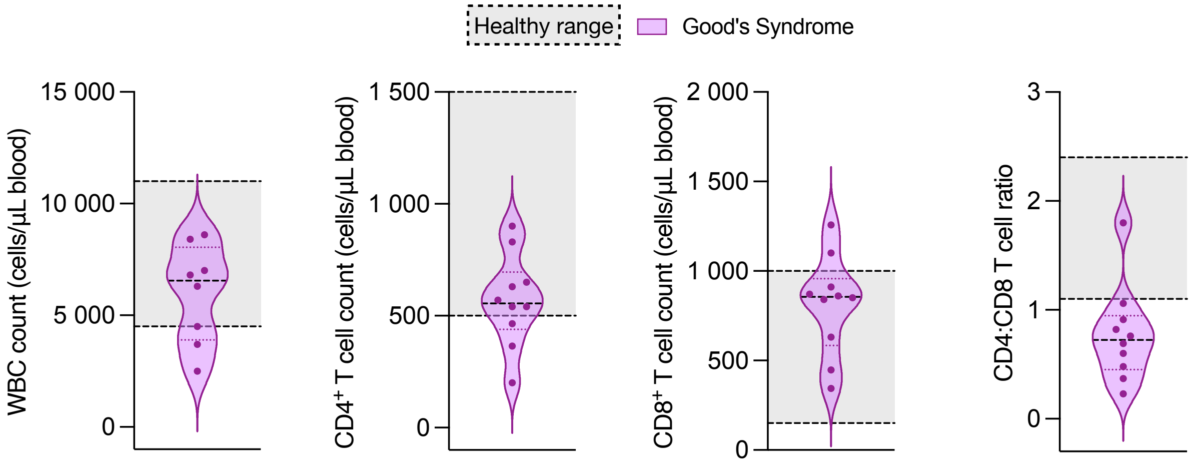

Supplementary Figure 2 – Circulating cell counts in Good's syndrome. Cell counts for Good's syndrome patients (white blood cells (WBC) (n = 8) B cells, T cells, CD4^{+} T cells, CD8^{+} T cells and the CD4/CD8 T cell ratio (n = 10) are graphed. Grey area represents normal healthy range.

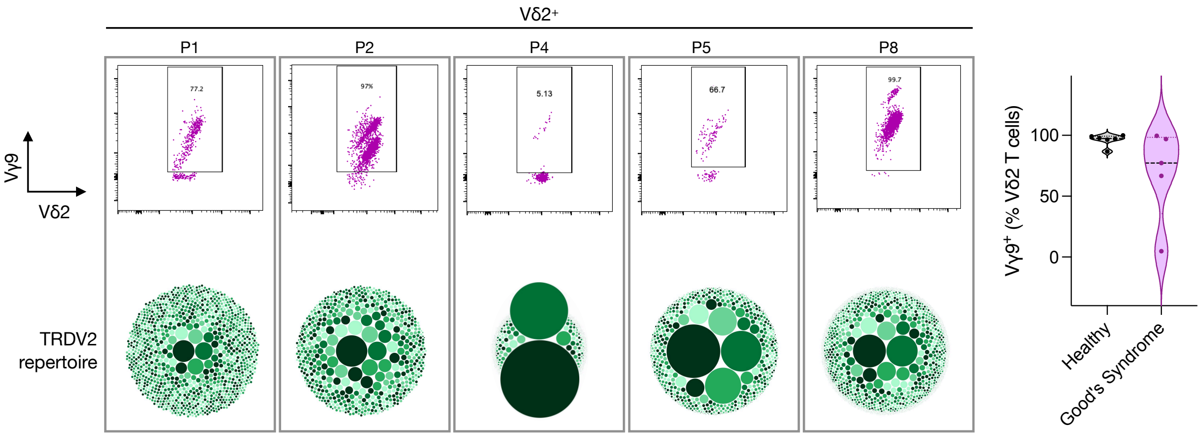

Supplementary Figure 3 – Vγ9 frequency among Vδ2^{+} γδ T cells. PBMC from Good's syndrome patients (n = 5) and healthy individuals (n = 6) were analysed by flow cytometry and Vδ2^{+} γδ T cells were gated on to assess frequency of Vγ9. Corresponding TRDV2 bubble plots are shown for comparison (derived from Figure 5, created using Flourish [https://flourish.studio]).

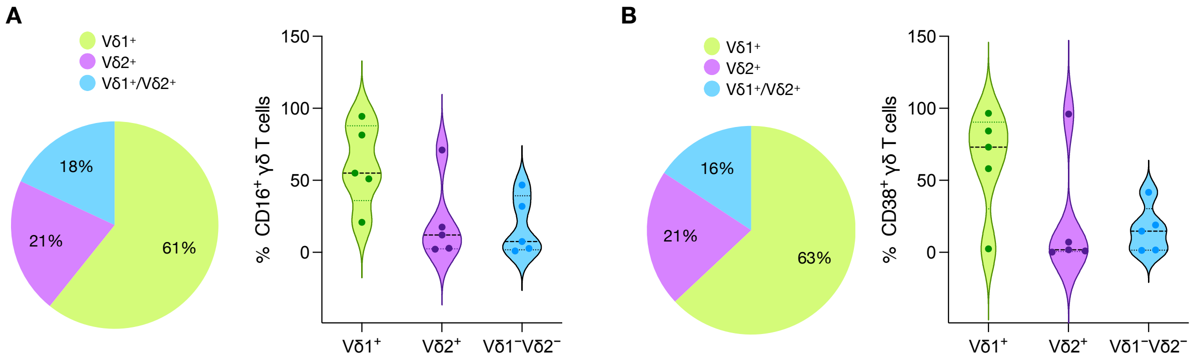

Supplementary Figure 4 – γδ T cell subset expression of CD16 and CD38. PBMC from Good's syndrome patients (n = 5) were analysed by flow cytometry and the proportion of γδ T cell subset cells contributing to the (A) $CD16^+$ and (B) $CD38^+$ γδ T cells were plotted and summarized in the pie graph.

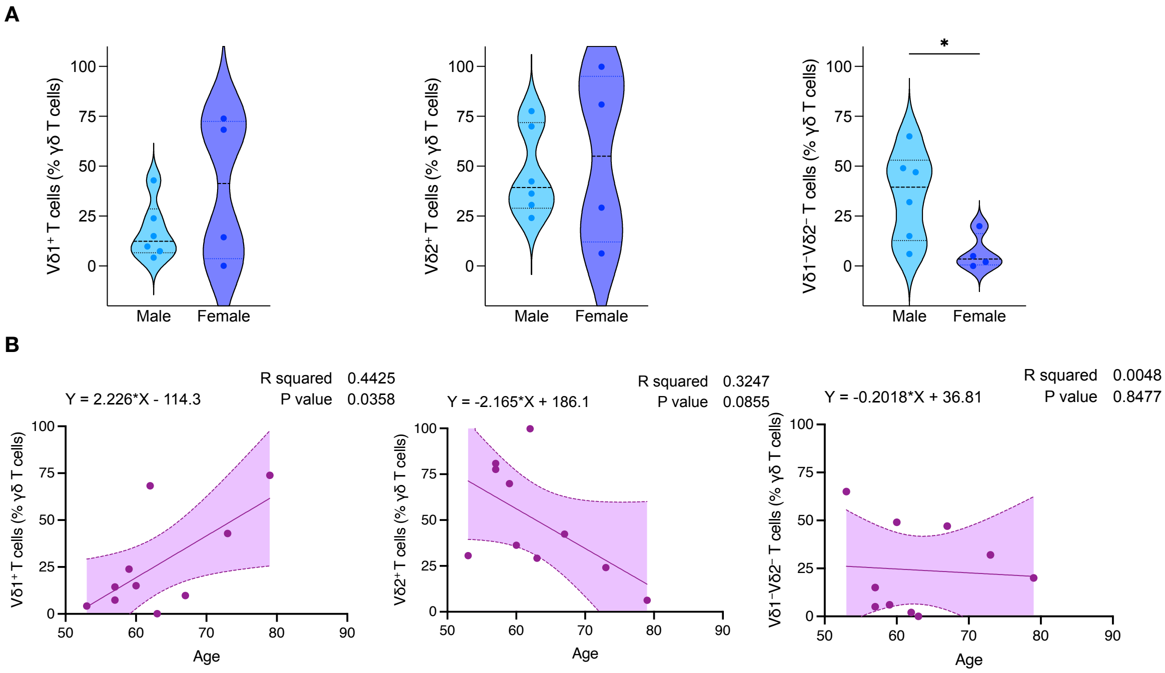

Supplementary Figure 5 – Impact of patient age and sex on the γδ T cell subsets in Good's syndrome patients. (**A**) The frequency of Good's syndrome patient (n = 10) γδ T cell subset frequency based on TCRδ repertoire analysis was plotted based on sex. Statistical significance was calculated using a two-tailed unpaired T test ,where **P* = 0.042. (**B**) The frequency of Good's syndrome patient (n = 10) γδ T cell subset frequency based on TCRδ repertoire analysis was plotted against age. Line represents simple linear regression with 95% confidence interval of the best fit line shown as shaded area between dashed lines. Statistical significance was calculated using simple linear regression (test for non-zero slope) with significance determined when $P < 0.05$ and R squared indicates goodness of fit.

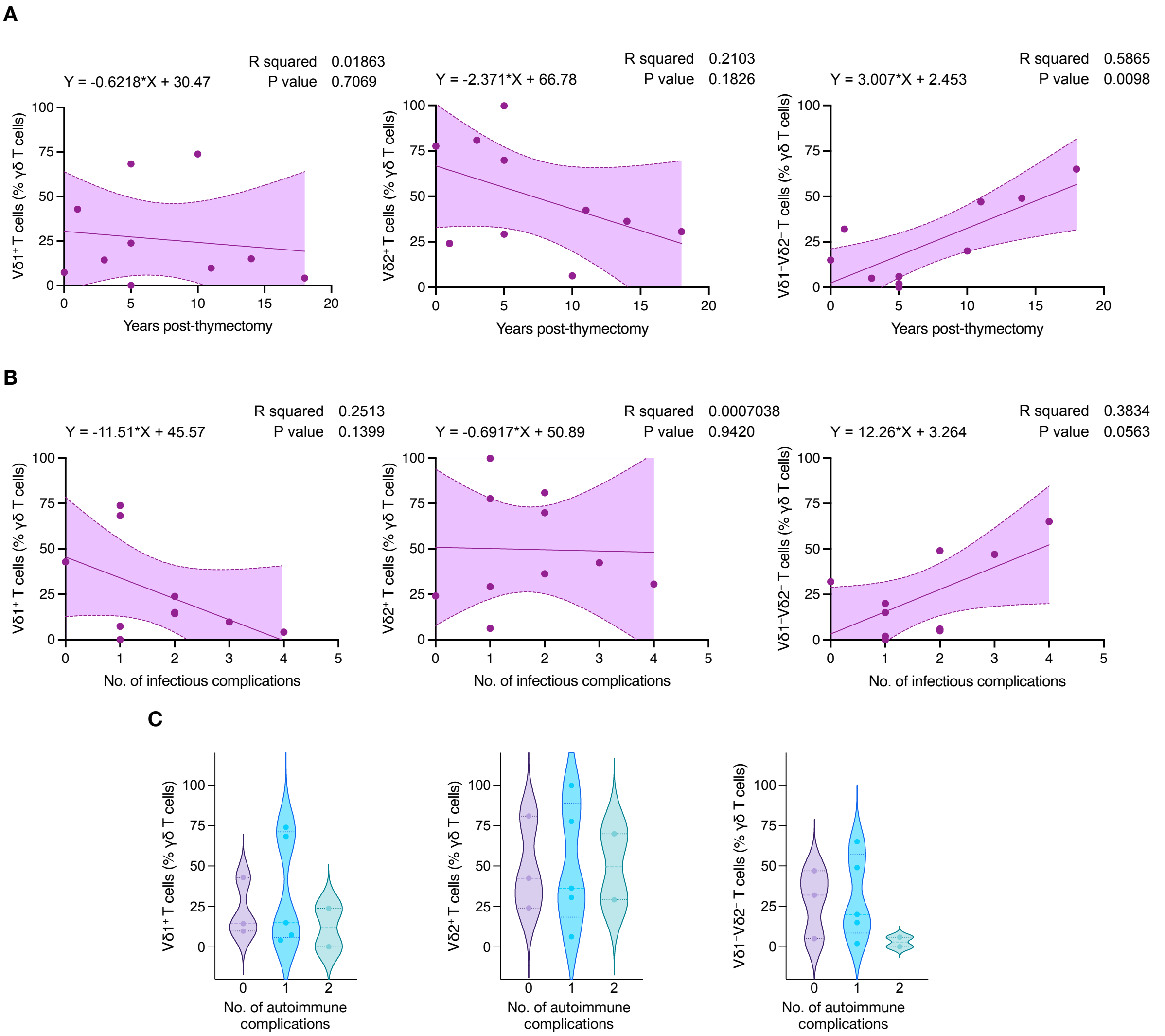

Supplementary Figure 6 – Impact of patient clinical variables on the γδ T cell subsets in Good's syndrome patients.

The frequency of Good's syndrome patient (n = 10) γδ T cell subset frequency based on TCRδ repertoire analysis was plotted against (**A**) years post-thymectomy, (**B**) number of infectious complications, and (**C**) number of autoimmune complications. (A–B) Line represents simple linear regression with 95% confidence interval of the best fit line shown as shaded area between dashed lines. Statistical significance was calculated using simple linear regression (test for non-zero slope) with significance determined when $P < 0.05$ and R squared indicates goodness of fit for (A–B) or using a one-way ANOVA with Tukey's multiple comparison test for (C).

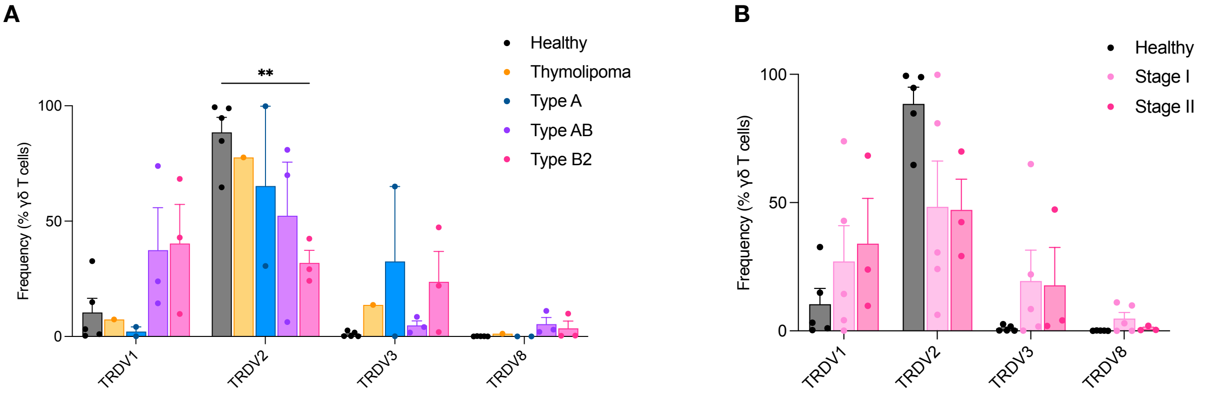

Supplementary Figure 7 – Impact of thymoma type and stage on the γδ T cell subsets in Good's syndrome patients.

The frequency of Good's syndrome patient (n = 10) γδ T cell subset frequency based on TCRδ repertoire analysis was grouped based on thymoma (**A**) type and (**B**) stage. Statistical significance was calculated using a one-way ANOVA with Tukey's multiple comparison test, where $^{**}P = 0.004$.

Supplementary Table 1 – Cell counts for Good's syndrome patients.

Donor ID	Total WBC count (cells/μL blood)	B cell count (cells/μL blood)	T cell count (cells/μL blood)	$CD4^+$ T cells (cells/μL blood)	$CD8^+$ T cells (cells/μL blood)	CD4:CD8 ratio	γδ T cells (% total T cells)
**P1**	4500	**10**^{L}	1670	630	910	**0.69**^{L}	9.6%
**P2**	8600	**0**^{L}	1400	540	850	**0.6**^{L}	5.3%
**P3**	**3700**^{L}	**0**^{L}	1250	**200**^{L}	870	**0.23**^{L}	**30%**^{H}
**P4**	8400	**10**^{L}	1900	**464**^{L}	**1258**^{H}	**0.37**^{L}	**13%**^{H}
**P5**	6300	**0**^{L}	1310	570	630	**0.91**^{L}	**37%**^{H}
**P6**	**2500**^{L}	**0**^{L}	1820	650	860	**0.76**^{L}	**11%**^{H}
**P7**	6800	**0**^{L}	1770	540	**1100**^{H}	**0.48**^{L}	9.0%
**P8**	ND	236	820	**365**^{L}	344	**0.82**^{L}	6.7%
**P9**	7000	**0**^{L}	1820	900	840	1.06	**16%**^{H}
**P10***	ND	154	1277	830	447	1.8	**11%**^{H}
**Normal reference range**	[4500–11 000]	[100–960]	[600–2500]	[500–1500]	[150–1000]	[1.1-2.4]	1–10%

Values in **bold** indicate outside normal healthy range, where ^{H} indicates higher and ^{L} indicates lower than healthy range. ND, not determined; WBC, white blood cell. *P10 clinical data has been previously published[1].

Supplementary Table 2 – Good's syndrome patient clinical and demographic details.

	P1	P2	P3	P4	P5	P6	P7	P8	P9	P10
BASELINE CHARATERISTICS										
**Year of birth**	1950	1971	1955	1964	1945	1965	1962	1961	1967	ND
**Death**	-	-	2023	2025	-	-	-	-	-	-
**Sex**	Male	Male	Male	Male	Female	Male	Female	Female	Female	Male
**Ethnicity**	Singaporean	Mauritian	Vietnamese	ND	Caucasian	Caucasian	Indian	Caucasian	Caucasian	Caucasian
THYMOMA										
**Diagnosis**	2024	2007	2012	2010	2015	2020	2020	2020	2022	57y
**Tumor type**	B2	A	B2	ND	AB	AB	B2	AB	AB	Thymolipoma
**Tumor stage**	I	I	II	ND	I	II	I	I	I	ND
CLINICAL FEATURES										
**Recurrent infection onset**	N/A	2010	2012	ND	2017	2020	2020	ND	2020	57y
**GS Diagnosis**	2024	2011	2012	2010	2017	2020	2020	2019	2023	57y
**Immuno-modulation**	nil	IVIg<br>SSZ	IVIg<br>SCIg	nil	PRED<br>CsA	IVIg<br>PRED	SCIg<br>HCQ<br>MMF	SCIg	PRED<br>IVIg<br>SCIg	MP/DXM<br>TPE<br>IVIg<br>Rituximab
**Major complications**<br>BE<br>Autoimmunity<br>Infections	Severe COVID19 requiring ICU	Reactive arthritis<br>Septic arthritis<br>*Salmonella* and *Mycoplasma*<br>*Pseudomonas* sinusitis<br>Chronic rhinosinusitis	BE<br>Recurrent cutaneous HSV<br>*Pseudomonas* bacteremia<br>Pulmonary nocardia	BE<br>Psoriasis<br>CMV hepatitis, gastritis and pneumonitis<br>Onycho-mycosis	Immune cytopenias<br>Chronic rhinosinusitis	Immune cytopenias<br>Lichen planus<br>Recurrent cutaneous HSV<br>Onychomycosis	Lichen planus<br>MAC pneumonia	Interstitial lung disease<br>Oral lichen planus<br>Chronic Norovirus enteropathy	Severe COVID19 with organizing pneumonia	Myasthenia gravis<br>Viral pneumonitis<br>*S. epidermidis* surgical site infection
CMV STATUS										
**IgG serostatus***	+	N/A	N/A	+	+	N/A	N/A	N/A	N/A	ND
**Viremia**	ND	−	+	+	ND	ND	ND	ND	+	ND

*Only applicable for patients not on Ig replacement therapy

BE, bronchiectasis; CMV, cytomegalovirus; CsA, cyclosporin; DXM, dexamethasone; GS, Good's syndrome; HCQ, Hydroxychloroquine; HSV, herpes simplex virus; ICU, intensive care unit; IVIg, intravenous immunoglobulin replacement; MAC, Mycobacterial Avium Complex; MMF, Mycophenolate mofetil; MP, Methylprednisolone; N/A, not applicable; ND, not determined/reported; PRED, Prednisolone; SCIg, subcutaneous immunoglobulin replacement; SSZ, Sulfasalazine; TPE, Therapeutic Plasma Exchange.

Supplementary Table 3 – Sample details for TRD repertoire analysis.

ID	Cohort	CMV status	Number of sorted γδ T cells	TRD sequence reads	Distinct CDR3
**HD1**	Healthy	+	69 575	317 743	995
**HD2**	Healthy	+	43 981	746 718	1 063
**HD3**	Healthy	+	108 200	314 472	7 032
**HD4**	Healthy	+	133 000	294 784	914
**HD5**	Healthy	+	208 000	584 659	2 552
**P1**	Good's syndrome	+	*Blood T1:* 469 700	1 048 260	36 907
			Blood T2: 108 000	585 130	20 812
			Thymoma: N/A	293 449	1 683
**P2**	Good's syndrome	ND	51 000	337 647	1 597
**P3**	Good's syndrome	+V	94 600	314 969	2 361
**P4**	Good's syndrome	+V	157 000	1 142 483	2 420
**P5**	Good's syndrome	+	966 000	1 622 335	4 180
**P6**	Good's syndrome	ND	332 300	1 480 150	4 466
**P7**	Good's syndrome	ND	100 600	1 144 855	4 158
**P8**	Good's syndrome	ND	170 048	473 115	2 587
**P9**	Good's syndrome	+V	302 000	1 110 606	7 071
**P10**	Good's syndrome	ND	105 969	296 351	5 566

ND, not determined; TRD, T cell receptor delta +V, positive CMV viral load +, positive CMV IgG serostatus.

Supplementary Table 4 – Top shared clonotypes between P9 and P10.

CDR3 sequences	TRDV	TRDJ	P9 frequency	P10 frequency
**ACDTGGWGISSLTAQLF**	TRDV2	TRDJ2	19.7%	18.3%
**ACDTGTGIRNGLI**	TRDV2	TRDJ1	23.3%	1.3%
**ACDKLRETDKLI**	TRDV2	TRDJ1	1.2%	5.6%
**ACDSLLGDKGLI**	TRDV2	TRDJ1	0.5%	1.4%
**ACFSVVLSDKLI**	TRDV2	TRDJ1	0.4%	1.0%

Supplementary Table 5 – Top expanded clonotypes for each patient.

ID	CDR3 sequences	TRDV	TRDJ	Frequency	Private/ shared
**P1**	ALPTFLRNPGGYARVDKLI	TRDV1	TRDJ1	1.3%	Private
	ALGEAPHYWGMRGRYTDKLI	TRDV1	TRDJ1	1.1%	Private
	ALGHSQGQWGIGKDTDKLI	TRDV1	TRDJ1	1.0%	Private
**P2**	ALDKPRWGTQTIKYTDKLI	TRDV3	TRDJ1	29.4%	Private
	ASLQRGILTDKLI	TRDV3	TRDJ1	28.9%	Private
**P3**	ALTSTGYTDKLI	TRDV3	TRDJ1	11.6%	Private
**P4**	ACDRRTGGYRDKLI	TRDV2	TRDJ1	21.1%	Private
	ACDPIFRTGGTQALTAQLF	TRDV2	TRDJ2	11.4%	Private
	ASHSGYWGINTDKLI	TRDV3	TRDJ1	9.6%	Private
**P5**	AYRRTESQFHFRPTTGGQPFRPPYTDKLI	TRDV8	TRDJ1	10.2%	Private
	ALGEQWPSDILGDTDKLI	TRDV1	TRDJ1	7.8%	Private
	ALGAGYGVSTDKLI	TRDV1	TRDJ1	6.6%	Private
**P6**	ACVPDVLPPLHKLI	TRDV2	TRDJ1	47.9%	Shared
**P7**	ALEDARWLVWWSGIQKYTDKLI	TRDV1	TRDJ1	38.7%	Private
**P8**	ACDTVGDKGDKLI	TRDV2	TRDJ1	10.8%	Shared
	ACDTVSPTQLGGYTDKLI	TRDV2	TRDJ1	9.7%	Private
**P9**	ACDTGTGIRNGLI	TRDV2	TRDJ1	23.3%	Shared
	ACDTGGWGISSLTAQLF	TRDV2	TRDJ2	19.7%	Shared
**P10**	ACDTGGWGISSLTAQLF	TRDV2	TRDJ2	18.3%	Shared

Shared indicates CDR3 sequence detected in at least one other individual at a read count of >50 to remove false-positives. Private indicates not detected in any other individuals in this cohort. TRDJ, T-cell receptor delta chain joining region; TRDV, T-cell receptor delta chain variable region.

References

1. Guevara-Hoyer K, Fuentes-Antras J, Calatayud Gastardi J, Sanchez-Ramon S. Immunodeficiency and thymoma in Good syndrome: Two sides of the same coin. *Immunol Lett* **231**, 11-17 (2021).
