# Supplementary material for: Oligoclonal expansion of atypical Vδ2− γδ T cells in Good’s Syndrome
Source: Nat Commun. 2026 Jun 11;17:7439. doi: 10.1038/s41467-026-74273-9 (PMC13407883; doi:10.1038/s41467-026-74273-9)
Supplement: Supplementary file 2 — Reporting Summary [file 41467_2026_74273_MOESM2_ESM.pdf]

nature portfolio

Corresponding author(s): Lauren J. Howson

Last updated by author(s): May 25, 2026

Reporting Summary

Nature Portfolio wishes to improve the reproducibility of the work that we publish. This form provides structure for consistency and transparency in reporting. For further information on Nature Portfolio policies, see our Editorial Policies and the Editorial Policy Checklist.

Statistics

For all statistical analyses, confirm that the following items are present in the figure legend, table legend, main text, or Methods section.

n/a	Confirmed	
☐	☒	The exact sample size (*n*) for each experimental group/condition, given as a discrete number and unit of measurement
☐	☒	A statement on whether measurements were taken from distinct samples or whether the same sample was measured repeatedly
☐	☒	The statistical test(s) used AND whether they are one- or two-sided<br>*Only common tests should be described solely by name; describe more complex techniques in the Methods section.*
☐	☒	A description of all covariates tested
☐	☒	A description of any assumptions or corrections, such as tests of normality and adjustment for multiple comparisons
☐	☒	A full description of the statistical parameters including central tendency (e.g. means) or other basic estimates (e.g. regression coefficient) AND variation (e.g. standard deviation) or associated estimates of uncertainty (e.g. confidence intervals)
☐	☒	For null hypothesis testing, the test statistic (e.g. *F*, *t*, *r*) with confidence intervals, effect sizes, degrees of freedom and *P* value noted<br>*Give P values as exact values whenever suitable.*
☒	☐	For Bayesian analysis, information on the choice of priors and Markov chain Monte Carlo settings
☒	☐	For hierarchical and complex designs, identification of the appropriate level for tests and full reporting of outcomes
☒	☐	Estimates of effect sizes (e.g. Cohen's *d*, Pearson's *r*), indicating how they were calculated

Our web collection on statistics for biologists contains articles on many of the points above.

Software and code

Policy information about availability of computer code

Data collection	SpectroFlo software (Cytek, v4)
Data analysis	FlowJo™ software (BD, v10)<br>Prism (GraphPad Software, v10)<br>iRweb (iRepertoire Inc.)<br>Flourish Software (Canva UK Operations Ltd)

For manuscripts utilizing custom algorithms or software that are central to the research but not yet described in published literature, software must be made available to editors and reviewers. We strongly encourage code deposition in a community repository (e.g. GitHub). See the Nature Portfolio guidelines for submitting code & software for further information.

Data

Policy information about availability of data

All manuscripts must include a data availability statement. This statement should provide the following information, where applicable:

- Accession codes, unique identifiers, or web links for publicly available datasets
- A description of any restrictions on data availability
- For clinical datasets or third party data, please ensure that the statement adheres to our policy

The γδ TCR repertoire raw sequence data supporting the findings of the study have been deposited in the NCBI Sequence Read Archive (SRA) under the BioProject

Accession Number: PRJNA1390857 [https://www.ncbi.nlm.nih.gov/bioproject/PRJNA1390857]. The RNA-seq raw sequence data has been deposited in the NCBI SRA under the BioProject Accession Number: PRJNA1446171 [https://www.ncbi.nlm.nih.gov/bioproject/PRJNA1446171]. Source data are provided with this article.

Research involving human participants, their data, or biological material

Policy information about studies with human participants or human data. See also policy information about sex, gender (identity/presentation), and sexual orientation and race, ethnicity and racism.

Reporting on sex and gender	Sex was self-reported for all study participants. All findings are applicable to both sexes. Sex was considered in the study design, with the healthy cohort being selected to represent a balanced sex ratio. The Good's syndrome cohort contained more males (60%) as selection for inclusion was not limited by sex (in order to obtain higher sample size), but clinical diagnosis. Sex-based analysis was performed (see Supplementary Fig. 5A).
Reporting on race, ethnicity, or other socially relevant groupings	Social groupings were not used for analysis in this study but ethnicity for patients has been provided in Supplementary Table 2.
Population characteristics	Age, sex covariates were collected for both healthy and Good's syndrome patients. Additional characteristics for patients included: age and years since Good's syndrome diagnosis, thymoma type and stage, infectious and autoimmune complications, immunomodulatory treatment, CMV status and death
Recruitment	Patient samples and clinical data were obtained from the Royal Melbourne Hospital and the Hospital Clínico San Carlos. Good's Syndrome was defined as thymoma associated with adult-onset immunodeficiency including hypogammaglobulinemia, consistent with the original case description and current commonly accepted criteria.<br><br>Healthy donors were recruited through the volunteer biospecimen donor registry at the Walter and Eliza Hall Institute of Medical Research (WEHI). Selection for inclusion in this study was based on obtaining a balance of ages and sexes across individuals with no known immunological or autoimmune conditions.<br><br>Potential sources of bias include the small cohort size inherent to this rare disease, inter-individual clinical heterogeneity, and recruitment across independent hospitals in inner-city settings, which may introduce socioeconomic or population-based biases. While these factors may contribute to variability in immune phenotypes, samples were processed and analysed using consistent approaches and the major findings were reproducible across patients and experimental batches.
Ethics oversight	Ethical approval for this study was granted by the Human Research Ethics Committees of Melbourne Health (project ID: 2009.162), WEHI (project ID: 25/26), and Hospital Clinic San Carlos (20/072-E). Written, informed consent was obtained from all participants, in accordance with the Declaration of Helsinki prior to their participation in the study.

Note that full information on the approval of the study protocol must also be provided in the manuscript.

Field-specific reporting

Please select the one below that is the best fit for your research. If you are not sure, read the appropriate sections before making your selection.

☒ Life sciences ☐ Behavioural & social sciences ☐ Ecological, evolutionary & environmental sciences

For a reference copy of the document with all sections, see nature.com/documents/nr-reporting-summary-flat.pdf

Life sciences study design

All studies must disclose on these points even when the disclosure is negative.

Sample size	Sample size for the Good's syndrome cohort was based on recruiting the maximum sample size available for inclusion in the study, ensuring statistical differences were able to be detected. The number of healthy donors included was matched to the number of Good's syndrome patients except for TCR repertoire analysis, which included 5 healthy donors as the repertoire of healthy individuals is already well described and reported in the literature.
Data exclusions	Data was excluded from samples contained high levels (>50%) dead cells.
Replication	Due to limitations in sample availability inherent to this rare disease cohort, technical replicates were not feasible on a per-individual basis. All experiments were therefore performed using the maximum number of independent biological samples available for each disease group, and all biological replicates were included in analyses unless excluded according to the predefined data exclusion criteria described above. To assess reproducibility and minimise potential batch effects, sorting, phenotyping, and TCR repertoire analyses were conducted across at least two independent experimental batches, with healthy controls and Good's syndrome samples run in parallel within each batch. Consistent findings were observed across experimental runs, supporting the robustness of the reported results.
Randomization	N/A, patients assigned to cohorts based on Good's syndrome diagnosis or healthy status.
Blinding	Investigators were not blinded to the group allocations of healthy donor and Good's syndrome cohorts for any experiments due to their separate sample acquisition pipelines. Analysis was not blinded but both healthy and Good's syndrome samples were batch analysed together with the same strategy applied to all samples within a batch.

Reporting for specific materials, systems and methods

We require information from authors about some types of materials, experimental systems and methods used in many studies. Here, indicate whether each material, system or method listed is relevant to your study. If you are not sure if a list item applies to your research, read the appropriate section before selecting a response.

Materials & experimental systems

n/a	Involved in the study
☐	☒ Antibodies
☒	☐ Eukaryotic cell lines
☒	☐ Palaeontology and archaeology
☒	☐ Animals and other organisms
☒	☐ Clinical data
☒	☐ Dual use research of concern
☒	☐ Plants

Methods

n/a	Involved in the study
☒	☐ ChIP-seq
☐	☒ Flow cytometry
☒	☐ MRI-based neuroimaging

Antibodies

Antibodies used

All antibodies used were commercially available and validated for specificity by the manufacturer. BD OptiBuild™ Brilliant Ultraviolet (BUV)615 anti-human CD27 (O323, 1:50), BD OptiBuild™ BUV805 anti-Human CD161 (DX12, 1:25), BD Horizon™ Brilliant Violet (BV)786 anti-human IFN-γ (4S.B3, 1:20), BD Horizon™ BV786 anti-human CD38 (HIT2, 1:25), and BD OptiBuild™ RealBlue (RB)613 anti-Human CD137 [4-1BB] (4B4-1, 1:25) were purchased from BD Biosciences. Alexa Fluor 700 anti-human CD28 (CD28.2, 1:25), allophycocyanin (APC) anti-human/mouse granzyme B (QA16A02, 1:10), BUV395 anti-human CD3 (SK7, 1:50), BV605 anti-human TCR Vα7.2 (3C10, 1:25), BV650 anti-human CX3CR1 (2A9-1, 1:25), PE/Dazzle 594 anti-human TNF-α (MAb11, 1:20), PE/Cyanine5 anti-human TCR Vγ9 (B3, 1:10) PE/Cyanine7 anti-human CD4 (SK3, 1:25), PerCP/Cyanine5.5 anti-human CD8 (SK1, 1:25) and PerCP/Cyanine5.5 anti-human CD16 (3G8, 1:25) were purchased from BioLegend. APC-Vio770 anti-human TCR Vδ1 (REA173, 1:10), Vioblue TCR Vδ2 (123R3, 1:100), and fluorescein isothiocyanate (FITC) anti-human TCRγ/δ (REA591, 1:10) were purchased from Miltenyi Biotec. . Immunoprofiling was performed using antibodies from the 25-Color Immunoprofiling Assay.

Validation

All antibodies used were commercially available and validated for specificity by the manufacturer as described on their websites:
https://www.biolegend.com/en-gb/kokd-validation
https://www.bdbiosciences.com/en-au/products/reagents/flow-cytometry-reagents/research-reagents/quality-and-reproducibility
https://www.miltenyibiotec.com/UN-en/products/macs-antibodies/recombinant-antibodies.html?query=:relevance:allCategoriesOR:10000737
https://cytekbio.com/products/25-color-immunoprofiling-kit?variant=39580454944804

Plants

Seed stocks

Report on the source of all seed stocks or other plant material used. If applicable, state the seed stock centre and catalogue number. If plant specimens were collected from the field, describe the collection location, date and sampling procedures.

Novel plant genotypes

Describe the methods by which all novel plant genotypes were produced. This includes those generated by transgenic approaches, gene editing, chemical/radiation-based mutagenesis and hybridization. For transgenic lines, describe the transformation method, the number of independent lines analyzed and the generation upon which experiments were performed. For gene-edited lines, describe the editor used, the endogenous sequence targeted for editing, the targeting guide RNA sequence (if applicable) and how the editor was applied.

Authentication

Describe any authentication procedures for each seed stock used or novel genotype generated. Describe any experiments used to assess the effect of a mutation and, where applicable, how potential secondary effects (e.g. second site T-DNA insertions, mosiacism, off-target gene editing) were examined.

Flow Cytometry

Plots

Confirm that:

☐ The axis labels state the marker and fluorochrome used (e.g. CD4-FITC).

☐ The axis scales are clearly visible. Include numbers along axes only for bottom left plot of group (a 'group' is an analysis of identical markers).

☒ All plots are contour plots with outliers or pseudocolor plots.

☒ A numerical value for number of cells or percentage (with statistics) is provided.

Methodology

Sample preparation	For flow cytometry, cells were thawed and stained with viability dye for 10 min at room temperature, followed by antibodies diluted in phosphate buffered saline (PBS) with 10% fetal bovine serum (FBS) (Sigma) for 20 min on ice. Cells were fixed and permeabilized using the eBioscience™ Foxp3 / Transcription Factor Staining Buffer Set (Invitrogen) according to manufacturer's instructions. Intracellular antibodies were then incubated for 45 min at room temperature diluted in permeabilization buffer.<br>For the 25-Color Immunoprofiling Assay, cFluor® Reagent Kit (Cytek), cells were thawed and stained according to manufacturer's instructions.
Instrument	Flow cytometry data was collected on a Cytek Aurora and the immunoprofiling assay data was acquired during cell sorting on a Cytek Aurora™ CS System
Software	Data were analyzed using FlowJo™ cell analysis software and aquired using SpectroFlo software (Cytek)
Cell population abundance	Up to 1 000 000 cells were sorted for each subset cell population.
Gating strategy	forward and side scatter (size), single cells, live, and CD3+ T cells and then gating on γδ TCR+ (with Vδ1+ and Vδ2+ subsets)

☒ Tick this box to confirm that a figure exemplifying the gating strategy is provided in the Supplementary Information.
