# Supplementary material for: Oligoclonal expansion of atypical Vδ2− γδ T cells in Good’s Syndrome
Source: Nat Commun. 2026 Jun 11;17:7439. doi: 10.1038/s41467-026-74273-9 (PMC13407883; doi:10.1038/s41467-026-74273-9)
Supplement: Supplementary file 3 — Transparent Peer Review file [file 41467_2026_74273_MOESM3_ESM.pdf]

nature portfolio

Peer Review File

Oligoclonal expansion of atypical Vδ2– γδ T cells in Good's Syndrome

Corresponding Author: Dr Lauren Howson

This file contains all reviewer reports in order by version, followed by all author rebuttals in order by version.

Version 0:

Reviewer comments:

Reviewer #1

(Remarks to the Author)
In this manuscript, Bandala-Sanchez, et al, explore the γδ T cell repertoire in Good's Syndrome. While recent studies have investigated γδ T cells in patients with inborn errors of immunity, to my knowledge this is the first time γδ T cells have been studied in Good's syndrome. The authors use flow cytometry and TCRδ/TRD repertoire analysis to study the γδ T cells in 5 healthy donors and 10 Good's syndrome patients.
The manuscript is well-written, and the figures are clearly presented and described in the legends.
I have some specific comments that should be addressed before publication.
1. My major criticism is that I feel the authors overemphasise the Vδ1/Vδ2neg expansions in the title and throughout to make the data seem more novel. Rather, it seems all adaptive γδ T cell subsets (including Vδ1s) are increased in number and/or activation state in patients with Good's Syndrome. This is to be expected as the adaptive γδ cells are contributing to adaptive immunity in patients with defects in conventional αβ T cells, as they do in other immunodeficiencies. While some patients have large Vδ3 and/or Vδ8 clonotypes, other patients have larger Vδ1 expansions. Related to this, the patients with the most distinct repertoires are reported in the main figure while those with more "conventional" repertoires are put in the supplementary figures- I would recommend all ten patients are included in the main figures for transparency.

I suspect the increases in the Vδ1 percentages are less significant than the increases in Vδ3/8 subsets because most healthy donors have a higher percentage of Vδ1 cells to start with, while healthy donors rarely have Vδ3/8 expansions. However, Figure 3A still shows an increase in Vδ1 T cells as a percent of γδ T cells. If the authors presented these data as total numbers of Vδ1+, Vδ2+, Vδ1/2neg cells per ul of blood in these donors (they should be able to calculate this as they have T cell counts in Supplementary Figure 2) they may see a significant expansion in absolute numbers in the Vδ1 compartment as well?

In the current manuscript, the focus on the expansion of Vδ1/2neg γδ T cells implies these cells are different to Vδ1 T cells in some way. To my knowledge, scRNAseq datasets have not shown transcriptional differences between Vδ1 and Vδ3/5/8 cells, rather γδ T cells expressing Vδ1/3/5/8 etc are clustered together distinct from pAg-reactive innate-like Vγ9Vδ2+ cells. Further evidence that all Vδ2neg subsets are functionally similar comes from Dechanet, 1999, and subsequent studies, where Vδ2neg subsets (including Vδ1, Vδ3, Vδ5, etc) expand in CMV; de Vries, 2023, where Vδ1 and Vδ3 both increase in checkpoint-blockade treated colorectal cancer; and Hunter, et al 2018, where all Vδ2neg cells (Vδ1, Vδ3, and even Vγ9negVδ2+ cells- see next comment) are increased and have similar phenotypes in the liver. Broadening the study to examine all adaptive subsets of γδ T cells in Good's Syndrome would provide a more complete picture of the γδ repertoire in this disease. However, it is possible that the increase in Vd1/2neg subsets could be a result of altered thymic output or distinct antigens in Good's syndrome, as the authors have suggested.

2. Since they have only sequenced TRD for these samples, the authors have ignored the possibility that some of the Vδ2 sequences may derive from Vγ9negVδ2+ cells, a subset that has been shown to align with other adaptive γδ T cell subsets and is expanded following CMV infection (Kaminski, 2020; Davey, 2018; McMurray, 2022). There is evidence for very large Vδ2 clonotypes in some patients (eg HD4, P4 and P6). This is unusual for Vγ9Vδ2 repertoires and could represent Vγ9negVδ2+ expansion rather than Vγ9Vδ2+ pAg-reactive cells. If a Vγ9 Ab was included in the staining panel they should investigate whether any of these donors/patients had large Vγ9negVδ2+ expansions, which would fit nicely with expansion of all non-Vγ9Vδ2+ adaptive γδ T cells in these patients.

3. Since Vδ2neg cells are known to be involved in CMV responses (Dechanet, 1999 and others), the authors should determine and report CMV serostatus in the healthy donors and patients. This could still be consistent with the increase in Vδ1/2neg subsets over time following thymectomy – as conventional T cells are affected in these patients following thymectomy, they may struggle to control CMV leading to expansion of some Vδ1 and Vδ1/2neg clonotypes.

4. The thymectomy data included in this manuscript is not particularly strong. They have thymus repertoire data from only one patient and obtained relatively few distinct CDR3 clonotypes in this analysis. This low diversity index suggests that the γδ T cells in the thymus are quite oligoclonal. However, other explanations are more likely. The repertoire analysis of this sample was done by isolating RNA from FFPE sections, and the quality of the isolated RNA may have been poor. Alternatively, there may have only been a small number of γδ T cells in the thymus section analysed. The authors should highlight these caveats. It would also strengthen the study if they could to obtain sequences from sorted γδ from thymectomy, and from multiple donors, if possible.

Minor points:
5. The observed clonal overlap of 9.6% in patients P9 and P10 is unusually high given the private nature of TRD clonotypes. The authors should comment on the sequences that were observed to be shared. Were these Vδ2 sequences or did they use other TRDV chains? Were these simple rearrangements with few N nucleotide additions? Or are the shared clonotypes the larger expanded clonotypes from one of the donors? If these samples were sequenced on the same chip this could be a sequencing/assignment error rather than true clonal overlap.

6. Expression of CD38 and CD16 is reported on total γδ T cells in Figure 2 rather than in specific γδ subsets. Presumably Vδ1 and Vδ2 antibodies were included in these staining panels, so expression of CD38 and CD16 could be delineated on different γδ subsets. In general, it would be interesting to attempt to relate the phenotyping data to the clonotyping data more in these donors.

7. In lines 177-179, the authors say TRDJ1 is associated with adult-derived γδ T cells, while TRDJ3 is associated with fetal-derived γδ T cells. While this was shown for Vγ9Vδ2 T cells, to my knowledge this has not been shown to be the case in other Vδ2neg T cell subsets.

8. In Figure 3E it is surprising there is not much response to anti-CD3/28 beads in the Vδ1 and Vδ1/2neg T cell subsets. Do you have any thoughts as to why this is the case? In other reports I believe Vδ1 cells in PBMC respond ex vivo to anti-CD3/28.

Overall this is an interesting study but I think broadening it to examine all adaptive-like γδ subsets in these patients, and in relation to CMV status, would improve the manuscript.

Reviewer #2

(Remarks to the Author)
This study provides the first analysis of γδ T cells in Good's syndrome. Using flow cytometry, functional assays, and TCRδ repertoire sequencing, the authors analyzed peripheral blood from 10 Good's syndrome patients and compared it with healthy controls. In one patient, γδ T cells from one thymoma tissue were additionally analyzed and compared with circulating cells. The study identifies expansion of γδ T cells in Good's syndrome, driven predominantly by Vd1 and Vd3 T cells. These cells display an activated, cytotoxic effector phenotype, characterized by high CD38, CD16, 4-1BB, CX3CR1, and granzyme B expression. Albeit the analysis and cohort is technically sound, the study is very descriptive and short in data. I have following concerns and suggestions to improve the study:

A) The study is very descriptive. While elevated gd T cell frequencies are obvious, it is unclear whether this is due to (i) direct thymic output due to the thymoma, (ii) compensatory proliferation to infections and loss of adaptive immunity (T cells and B cells, (iii) or direct expansion to tumor antigens. Therefore, the authors should perform transcriptomic analysis and/or analyze proliferative markers and cell markers for thymic output to better elucidate on potential causes.
B) The functional consequence on clinical output and phenotypes on the observed changes in the gd T cell compartment are not addressed in detail. Can the authors provide more details on the clinical features of the individual patients. For instance, not in every patient an expansion of Vd3 T cells is evident.
C) The TCR-seq identified several expanded clones, but is extremely descriptive. What is the reactivity of theses clones? Can the authors model thymoma in vitro to assess potential changes in phenotypes and TCR repertoires from healthy blood cells upon exposure to the in vitro thymoma to assess the direct effect of the tumor on gd T cells.
D) The authors point out that expansion of Vd3 T cell and Vd8 T cells are evident. Given the frequencies among all gd T cells and within individual donors, I would rather state that Vd1, Vd3 and Vd8 T cells are elevated in these patients. Also, I would not use expansion, as it is not clear whether the cells are really expanding or just show a higher abundance due to absence of other immune cells.
E) In Figure 2 can the authors also plot gd T cells, Vd2 T cells, and Vd1 T cells among all lymphocytes, which may show only abundance of Vd1 and Vd3 T cells in Good's syndrome. Also in the related supplement absolute cell counts for gd T cells should be shown.

Reviewer #3

(Remarks to the Author)
The authors use the rare Good's syndrome to study Tgd development in humans. This is an elegant approach to better characterize this otherwise understudied cell population.

Major

Functional insight is limited. Could the authors apply some additional bioinformatics tools such as Monocle analysis to better understand intrapatient transcriptomic heterogeneity and potentially developmental trajectories.

Version 1:

Reviewer comments:

Reviewer #1

(Remarks to the Author)
I have read the revised manuscript submitted by Bandala-Sanchez, et al. The authors have addressed my comments and concerns, and this has improved the study. They provide new analyses showing that in addition to increased Vd1/2negative cells in Good's Syndrome, Vd1 T cells are also increased. This is especially clear on the plots showing total cell counts. Their decision to change in the title based on these analyses is justified and will prevent confusion in the field.

The new Supplementary Table 3 including more information about the patients and their CMV status is useful. However, the legend for this table is a bit confusing at present. They need to clarify that "+" means CMV seropositive while "V+" means CMV viremia.

The new RNAseq data and analyses bolster the paper and show that different gd T cell subsets broadly maintain their normal phenotypes in Good's syndrome, although there are some interesting differences highlighted. However, these differences have not been validated at the protein level so the significance of these transcriptional changes should not be overstated.

While the authors have reassured me that the large clonal overlap between patients 9 and 10 is not a result of a sequencing error and could be due to a relatively simple, common rearrangement, I am concerned by some of the other clonotypes listed as shared between donors in the new Supplementary Table 5. Some of these clonotypes are extremely complex and I can tell they contain many N nucleotide additions (though without the actual CDR3 nucleotide sequence I cannot say exactly how many) and are therefore extremely unlikely to be generated in two individual donors. One example of this is the sequence from donor P5: AYRRTESQFHFRPTTGGQPFRPPYTDKLIF. The 20 amino acid (60 nucleotide) sequence RTESQFHFRPTTGGQPFRPP must be derived from D gene segments and N nucleotide addition. While TCRd often uses two TRDD gene segments, these segments are relatively short and this still leaves many N nucleotides added (for this sequence I estimate at minimum 40-50 N nucleotides). I would encourage the authors to look carefully at whether the two donors who share this sequence were sequenced on the same chip and if there is any chance that the sequence could have been misassigned, or if contamination could have occurred at another point in the sample preparation. I only highlight this because there is a misconception in the field that it is common/likely to identify shared gd TCR sequences between donors (perpetuated by sequencing errors or sample contamination in several published studies). However, the probability of generating a shared sequence with 40 N nucleotides is $1:4^{40}$ which is $1:1x10^{24}$ (approximately 27 billion times larger than the total number of cells in the human body), hence this is extremely unlikely/impossible. The sequence from donor P7 also looks complex and likely to contain many N nucleotides, as do several others.

Reviewer #2

(Remarks to the Author)
The authors have addressed all concerns in an excellent way.

Reviewer #3

(Remarks to the Author)
The authors have sufficiently responded to the reviewers questions and comments.

Open Access This Peer Review File is licensed under a Creative Commons Attribution 4.0 International License, which permits use, sharing, adaptation, distribution and reproduction in any medium or format, as long as you give appropriate credit to the original author(s) and the source, provide a link to the Creative Commons license, and indicate if changes were made.
In cases where reviewers are anonymous, credit should be given to 'Anonymous Referee' and the source.
The images or other third party material in this Peer Review File are included in the article's Creative Commons license, unless indicated otherwise in a credit line to the material. If material is not included in the article's Creative Commons license and your intended use is not permitted by statutory regulation or exceeds the permitted use, you will need to obtain permission directly from the copyright holder.
To view a copy of this license, visit https://creativecommons.org/licenses/by/4.0/

Point-by-Point Response to Reviewers' Comments

<u>Reviewer #1</u>

We are thankful for Reviewer 1's careful reading and thoughtful feedback on our manuscript, and that they state: ***"The manuscript is well-written, and the figures are clearly presented and described in the legends... To my knowledge this is the first time γδ T cells have been studied in Good's syndrome... Overall this is an interesting study".***

Please see below for our responses to specific points raised.

Comment 1A:

My major criticism is that I feel the authors overemphasise the Vδ1/Vδ2neg expansions in the title and throughout to make the data seem more novel. Rather, it seems all adaptive γδ T cell subsets (including Vδ1s) are increased in number and/or activation state in patients with Good's Syndrome. This is to be expected as the adaptive γδ cells are contributing to adaptive immunity in patients with defects in conventional αβ T cells, as they do in other immunodeficiencies. While some patients have large Vδ3 and/or Vδ8 clonotypes, other patients have larger Vδ1 expansions. Related to this, the patients with the most distinct repertoires are reported in the main figure while those with more "conventional" repertoires are put in the supplementary figures - I would recommend all ten patients are included in the main figures for transparency.

<u>Response:</u>

We appreciate Reviewer 1's perspective regarding the overall narrative regarding the alterations in the γδ T cells in Good's syndrome. We agree that whilst emphasizing the novelties related to Good's syndrome compared to other immunodeficiencies was a focus of our manuscript, it is true that in certain patients the Vδ1 cells were also altered and participating in the adaptive γδ T cell response.

To address this, we have changed the title of the manuscript to more broadly encompass the entire Vδ2^{-} compartment, and it now reads:

"Oligoclonal expansion of atypical Vδ2^{-} γδ T cells in Good's Syndrome"

We have also changed subheadings to encompass results relating to the entire Vδ2^{-} γδ T cell compartment:

(**page 5, line 128**):

> **"The Vδ2^{-} γδ T-cell subset is expanded, activated and cytotoxic in Good's syndrome"**

(**page 6, line 165**):

> **"TCRδ repertoire analysis reveals the Vδ2^{-} γδ T-cell compartment in Good's syndrome is primarily composed of Vδ1^{+}, Vδ3^{+} and Vδ8^{+} cells"**

As suggested, we have moved all 10 patient TCRδ chain usage and TCRδ repertoire bubble plots to the main for **Figure 4A:**

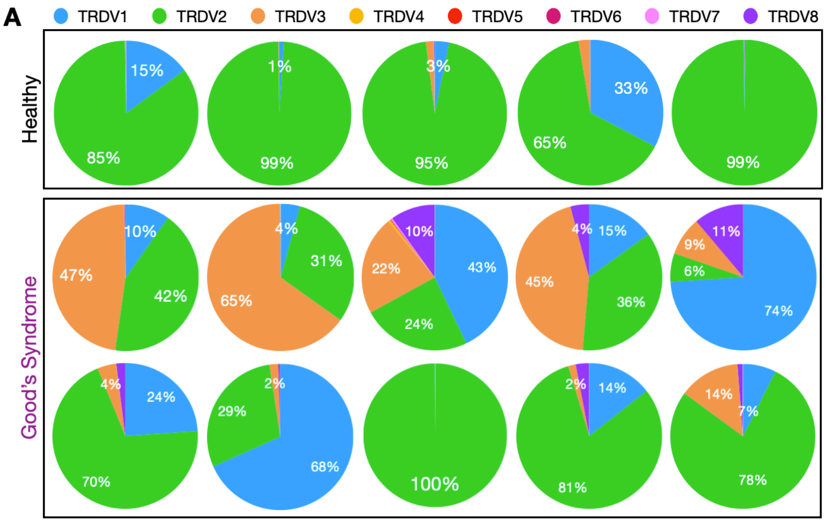

And **Figure 5A:**

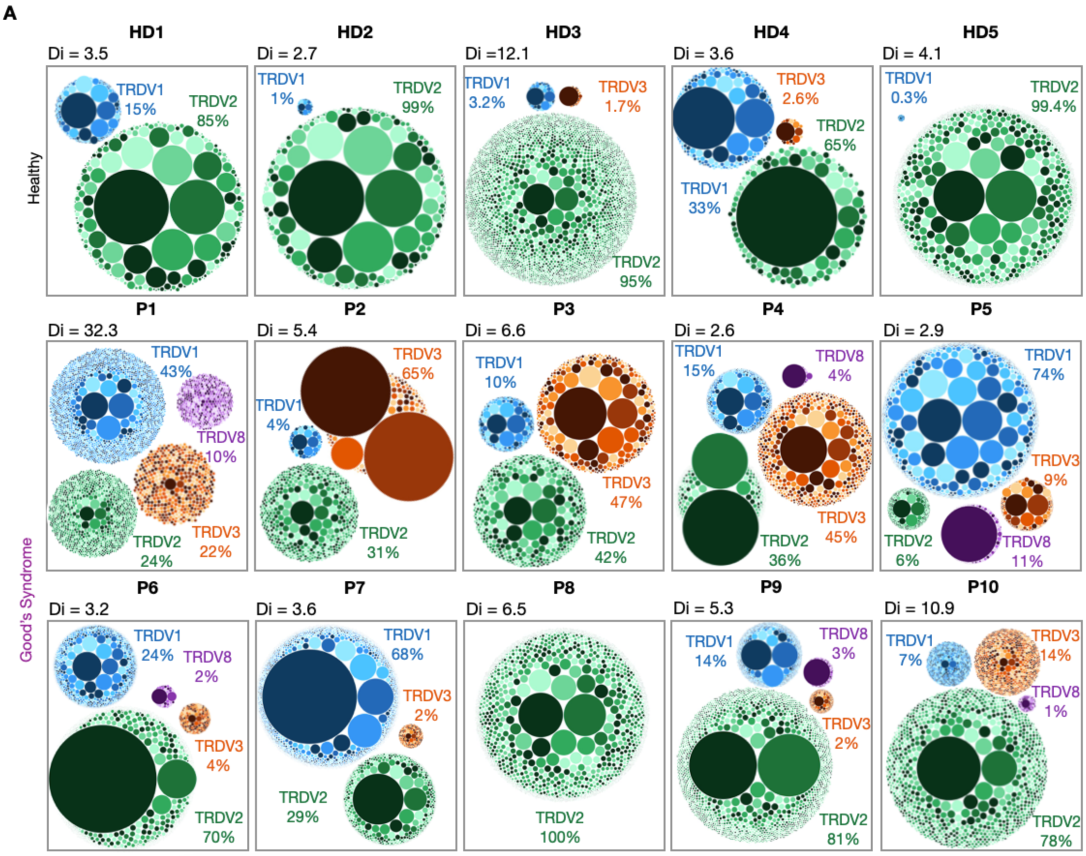

Comment 1B:

I suspect the increases in the Vδ1 percentages are less significant than the increases in Vδ3/8 subsets because most healthy donors have a higher percentage of Vδ1 cells to start with, while healthy donors rarely have Vδ3/8 expansions. However, Figure 3A still shows an

increase in Vδ1 T cells as a percent of γδ T cells. If the authors presented these data as total numbers of Vδ1+, Vδ2+, Vδ1/2neg cells per ul of blood in these donors (they should be able to calculate this as they have T cell counts in Supplementary Figure 2) they may see a significant expansion in absolute numbers in the Vδ1 compartment as well?

<u>Response:</u>

We thank Reviewer 1 for this suggestion, and we have now calculated the total counts for the γδ T cell subsets to provide clarity around whether these alterations in the compartments are true expansions of specific γδ T cell subsets.

This new data is now presented in **Figure 3B**:

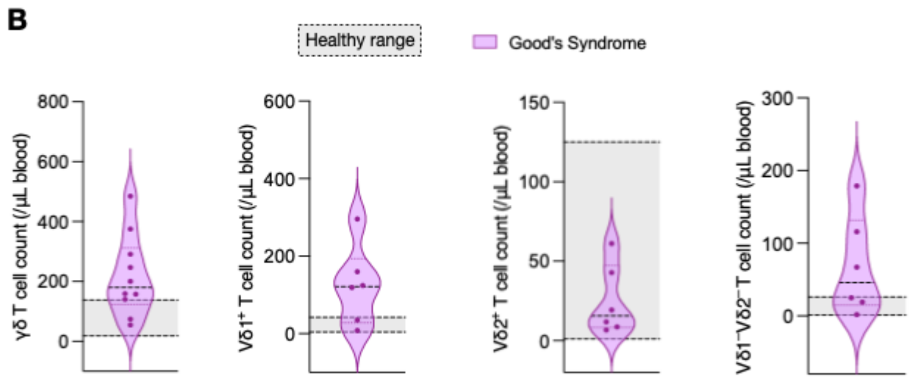

We have also updated the main text to include discussion of the changes in both Vδ1 and Vδ1⁻Vδ2⁻ subsets (**page 6, line 139-142**):

> "When γδ T cells were assessed as absolute counts, the majority of Good's syndrome patients exhibited elevated numbers of γδ T cells beyond that of the healthy range (**Figure 3B**). This expansion was driven by increased numbers of both Vδ1⁺ and Vδ1⁻Vδ2⁻ subsets, whereas Vδ2⁺ γδ T cell counts remained within the normal healthy range across all patients"

Comment 1C:

In the current manuscript, the focus on the expansion of Vδ1/2neg γδ T cells implies these cells are different to Vδ1 T cells in some way. To my knowledge, scRNAseq datasets have not shown transcriptional differences between Vδ1 and Vδ3/5/8 cells, rather γδ T cells expressing Vδ1/3/5/8 etc are clustered together distinct from pAg-reactive innate-like Vγ9Vδ2+ cells. Further evidence that all Vδ2neg subsets are functionally similar comes from Dechanet, 1999, and subsequent studies, where Vδ2neg subsets (including Vδ1, Vδ3, Vδ5, etc) expand in CMV; de Vries, 2023, where Vδ1 and Vδ3 both increase in checkpoint-blockade treated colorectal cancer; and Hunter, et al 2018, where all Vδ2neg cells (Vδ1, Vδ3, and even Vγ9negVδ2+ cells- see next comment) are increased and have similar phenotypes in the liver. Broadening the study to examine all adaptive subsets of γδ T cells in Good's Syndrome would provide a more complete picture of the γδ repertoire in this disease.

However, it is possible that the increase in Vd1/2neg subsets could be a result of altered thymic output or distinct antigens in Good's syndrome, as the authors have suggested.

<u>Response:</u>

To more broadly examine all changes in γδ T cell subsets we have updated the supplementary figures to include both Vδ1 and Vδ2 subsets in frequency analysis comparing the impact of the baseline and clinical patient characteristics on frequency of γδ T cell subsets.

This new analysis is now presented in **Supplementary Figure 3**:

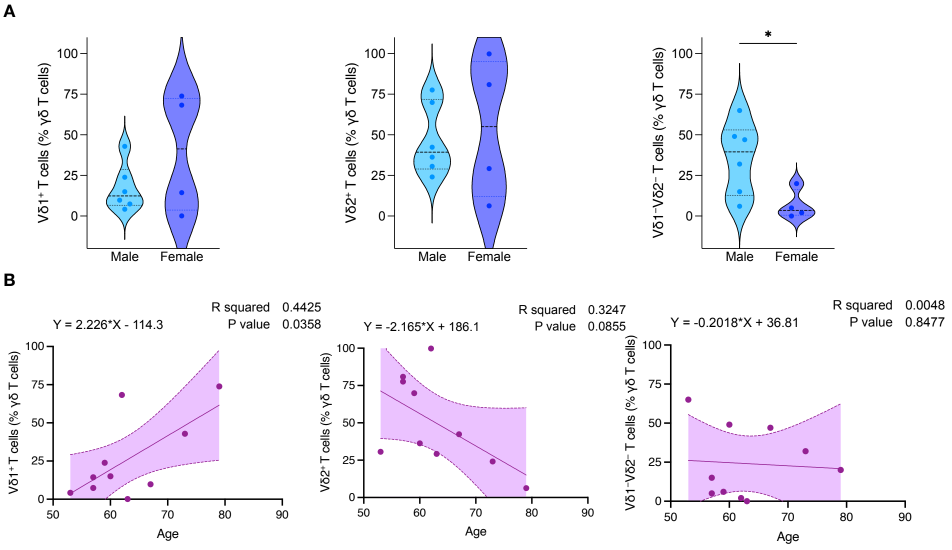

and **Supplementary Figure 4**:

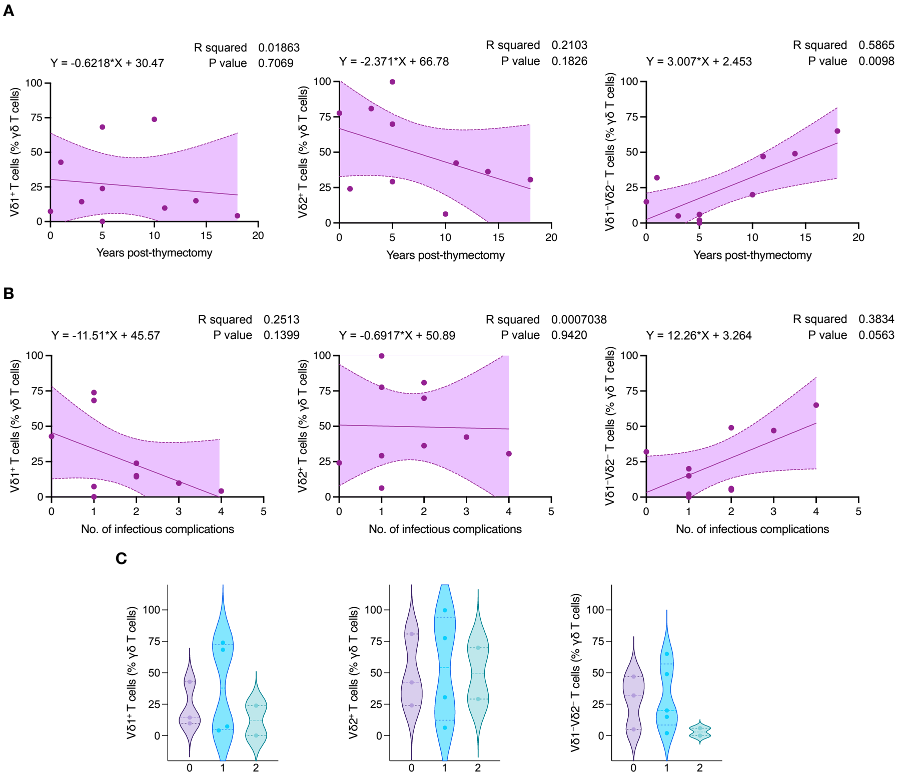

We have also performed an RNAseq experiment on sorted healthy and patient γδ T cell subsets to address the question of functional state and transcriptional profiles of all γδ subsets in GS.

This new data is presented in **Figure 7**:

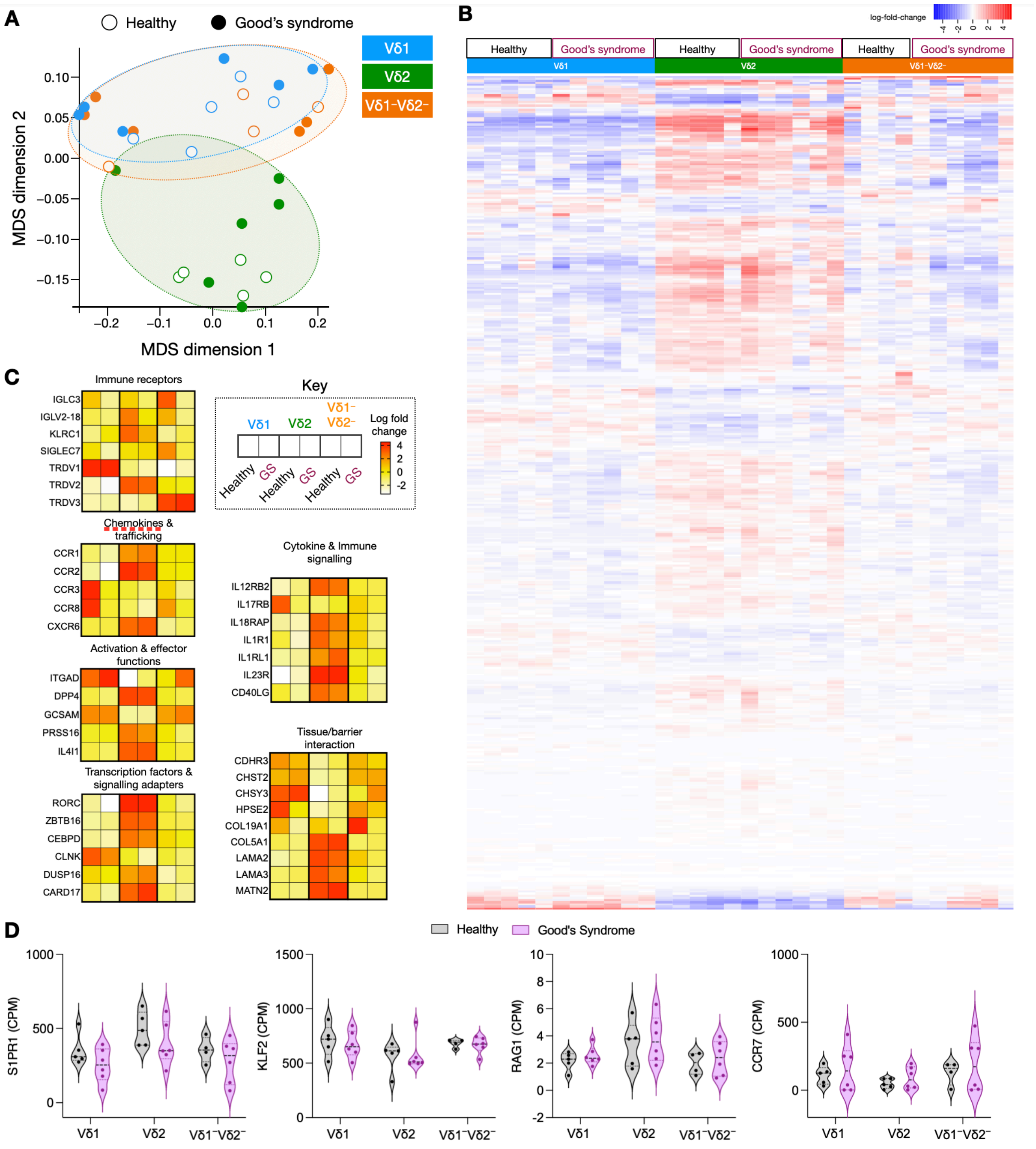

With the following also added to the main text (**page 9-10, line 252-268**):

> "To determine whether the γδ T cell subsets in Good's syndrome are functionally distinct and comparable to those observed in healthy individuals, we performed RNA sequencing on *ex vivo* sorted subsets. Consistent with prior reports Vδ2^{+} and Vδ2^{-} γδ T cells exhibited clear transcriptional separation, which was largely maintained between healthy individuals and Good's syndrome patients (**Figure 7A–B**). However, Vδ2^{+} cells from Good's syndrome patients

showed increased dispersion towards the Vδ2⁻ transcriptional space, suggesting partial convergence towards an adaptive-like state (**Figure 7A)**, in line with the expanded Vδ2⁺Vγ9⁻ population we observed in certain patients (**Supplementary Figure 3**).

At the gene level, differential expression analysis revealed selective alterations in immune-related pathways. Vδ1⁺ γδ T cells from Good's syndrome patients displayed reduced expression of *CCR3*, *CCR8*, *IL17RB* and *HPSE2* consistent with altered migratory and effector potential. In Vδ1⁻Vδ2⁻ subsets, increased *ITGAD* expression alongside reduced *IGLC3*, *SIGLEC7*, *CCR8* and *COL19A1 expression* suggests shifts in adhesion, immune regulation, and tissue interaction programs. Collectively, these data indicate that while core subset-defining transcriptional programs are preserved, Good's syndrome is associated with targeted rewiring of immune pathways, suggesting that γδ T cells retain lineage identity but undergo context-dependent functional adaptation.

Comment 2

Since they have only sequenced TRD for these samples, the authors have ignored the possibility that some of the Vδ2 sequences may derive from Vγ9negVδ2+ cells, a subset that has been shown to align with other adaptive γδ T cell subsets and is expanded following CMV infection (Kaminski, 2020; Davey, 2018; McMurray, 2022). There is evidence for very large Vδ2 clonotypes in some patients (eg HD4, P4 and P6). This is unusual for Vγ9Vδ2 repertoires and could represent Vγ9negVδ2+ expansion rather than Vγ9Vδ2+ pAg-reactive cells. If a Vγ9 Ab was included in the staining panel they should investigate whether any of these donors/patients had large Vγ9negVδ2+ expansions, which would fit nicely with expansion of all non-Vγ9Vδ2+ adaptive γδ T cells in these patients.

<u>Response:</u>

We thank reviewer 1 for this suggestion, as it is true that we do not explore the possibility that some of the Vδ2⁺ could be composed of the more adaptive-like Vγ9 negative. Based on indirect evidence of our HMBPP stimulation (Figure 2F), it does appear that the Good's syndrome patients have a smaller proportion of their Vδ2⁺ subset that responds to this ligand, suggesting a high frequency of the adaptive Vγ9⁻ cells in this compartment.

Unfortunately, we did not include the Vγ9 antibody in the original staining panel. However, we have performed additional flow cytometry staining experiments on the samples in which we still have material available to explore this possibility.

This new data is presented in **Supplementary Figure 3**:

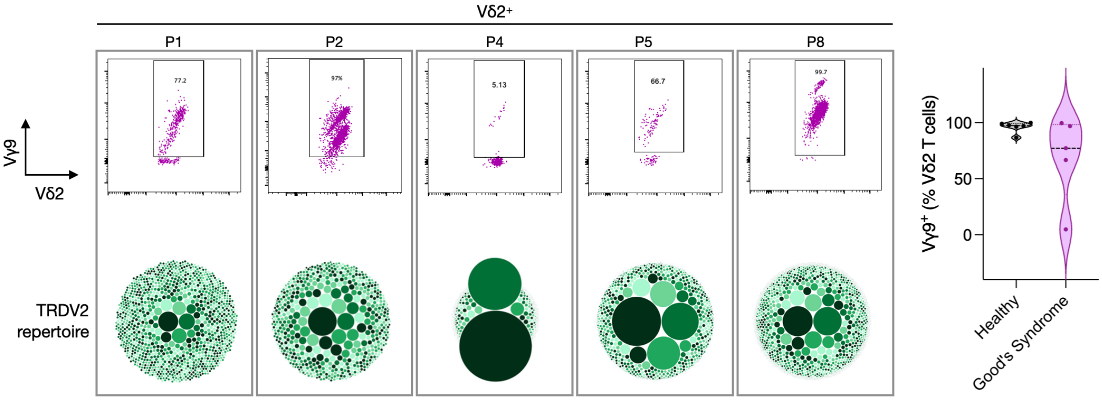

Comment 3

Since Vδ2neg cells are known to be involved in CMV responses (Dechanet, 1999 and others), the authors should determine and report CMV serostatus in the healthy donors and patients. This could still be consistent with the increase in Vδ1/2neg subsets over time following thymectomy – as conventional T cells are affected in these patients following thymectomy, they may struggle to control CMV leading to expansion of some Vδ1 and Vδ1/2neg clonotypes.

Response:

We agree that CMV status is an important variable when assessing γδ T cell composition and phenotype. As suggested, we have serotyped the healthy donors and now included the information provided in **Supplementary Table 3**.

ID	Cohort	CMV status	Number of sorted γδ T cells	TRD sequence reads	Distinct CDR3
**HD1**	Healthy	+	69 575	317 743	995
**HD2**	Healthy	+	43 981	746 718	1 063
**HD3**	Healthy	+	108 200	314 472	7 032
**HD4**	Healthy	+	133 000	294 784	914
**HD5**	Healthy	+	208 000	584 659	2 552
**P1**	Good's syndrome	+	*Blood T1:* 469 700	1 048 260	36 907
			Blood T2: 108 000	585 130	20 812
			Thymoma: N/A	293 449	1 683
**P2**	Good's syndrome	ND	51 000	337 647	1 597
**P3**	Good's syndrome	+V	94 600	314 969	2 361
**P4**	Good's syndrome	+V	157 000	1 142 483	2 420
**P5**	Good's syndrome	+	966 000	1 622 335	4 180
**P6**	Good's syndrome	ND	332 300	1 480 150	4 466
**P7**	Good's syndrome	ND	100 600	1 144 855	4 158
**P8**	Good's syndrome	ND	170 048	473 115	2 587
**P9**	Good's syndrome	+V	302 000	1 110 606	7 071
**P10**	Good's syndrome	ND	105 969	296 351	5 566

ND, not determined; TRD, T cell receptor delta V+, positive CMV viral loads.

For the Good's syndrome cohort, CMV serology is not interpretable in six patients due to ongoing immunoglobulin (Ig) replacement therapy. Where patients were not receiving Ig replacement, CMV serostatus has been included. In addition, CMV viral load testing was performed in selected patients with clinical suspicion of CMV disease as part of routine care, and these results are also now reported in **Supplementary Table 2**:

	P1	P2	P3	P4	P5	P6	P7	P8	P9	P10
BASELINE CHARATERISTICS										
**Year of birth**	1950	1971	1955	1964	1945	1965	1962	1961	1967	ND
**Death**	-	-	2023	2025	-	-	-	-	-	-
**Sex**	Male	Male	Male	Male	Female	Male	Female	Female	Female	Male
**Ethnicity**	Singaporea n	Mauritian	Vietnamese	ND	Caucasian	Caucasian	Indian	Caucasian	Caucasian	Caucasian
THYMOMA										
**Diagnosis**	2024	2007	2012	2010	2015	2020	2020	2020	2022	57y
**Tumor type**	B2	A	B2	ND	AB	AB	B2	AB	AB	Thymolipo ma
**Tumor stage**	I	I	II	ND	I	II	I	I	I	ND
CLINICAL FEATURES										
**Recurrent infection onset**	N/A	2010	2012	ND	2017	2020	2020	ND	2020	57y
**GS Diagnosis**	2024	2011	2012	2010	2017	2020	2020	2019	2023	57y
**Immuno-modulation**	nil	IVIg SSZ	IVIg SCIg	nil	PRED CsA	IVIg PRED	SCIg HCQ MMF	SCIg	PRED IVIg SCIg	MP/DXM TPE IVIg Rituximab
**Major complications** BE Autoimmunity Infections	Severe COVID19 requiring ICU	Reactive arthritis Septic arthritis: *Salmonella* and *Mycoplasma* *Pseudomonas* sinusitis Chronic rhinosinusitis	BE Recurrent cutaneous HSV *Pseudomonas* bacteremia Pulmonary nocardia	BE Psoriasis CMV hepatitis, gastritis and pneumonitis Onychomycosis	Immune cytopenias Chronic rhinosinusitis	Immune cytopenias Lichen planus Recurrent cutaneous HSV Onychomycosis	Lichen planus MAC pneumonia	Interstitial lung disease Oral lichen planus Enteropathy with norovirus	Severe COVID19 with organizing pneumonia	Myasthenia gravis Viral-type pneumonia *S. epidermidis* surgical site infection
CMV STATUS										
**IgG serostatus***	+	N/A	N/A	+	+	N/A	N/A	N/A	N/A	ND
**Viremia**	ND	−	+	+	ND	ND	ND	ND	+	ND

*Only applicable for patients not on Ig replacement therapy

BE, bronchiectasis; CMV, cytomegalovirus; CsA, cyclosporin; DXM, dexamethasone; GS, Good's syndrome; HCQ, Hydroxychloroquine; HSV, herpes simplex virus; ICU, intensive care unit; IVIg, intravenous immunoglobulin replacement; MAC, Mycobacterial Avium Complex; MMF, Mycophenolate mofetil; MP, Methylprednisolone; N/A, not applicable; ND, not determined/reported; PRED, Prednisolone; SCIg, subcutaneous immunoglobulin replacement; SSZ, Sulfasalazine; TPE, Therapeutic Plasma Exchange.

We have also updated the main text discussion to highlight the CMV status of the cohorts and its potential implication on the results (**page 11–12, line 305–313**):

> "We found that the expansion of the γδ T cell compartment in Good's syndrome was primarily driven by an increased frequency of $V\delta2^-$ γδ T cells. This could, in part, be due to the patients' CMV status as it is one of the most well characterized drivers of $V\delta2^-$ γδ T cell clonal expansion. Of the 10 patients in the Good's syndrome cohort, 3 have CMV viremia and 2 are seropositive for CMV, the remaining 5 were on Ig replacement so their serostatus could not be determined. However, in contrast to CVID with CMV disease, where the predominant and activated subset is $V\delta1^+$ cells[17], both $V\delta1^+$ and $V\delta1^-V\delta2^-$ subsets were significantly expanded in Good's syndrome. Notably, it was the $V\delta1^-V\delta2^-$ subset that exhibited an effector-like phenotype with elevated cytotoxic potential. These findings suggest that the mechanisms driving γδ T-cell subset skewing differ between CVID/CMV and Good's syndrome."

Comment 4

The thymectomy data included in this manuscript is not particularly strong. They have thymus repertoire data from only one patient and obtained relatively few distinct CDR3 clonotypes in this analysis. This low diversity index suggests that the γδ T cells in the thymus are quite oligoclonal. However, other explanations are more likely. The repertoire analysis of this sample was done by isolating RNA from FFPE sections, and the quality of the isolated RNA may have been poor. Alternatively, there may have only been a small number of γδ T cells in the thymus section analysed. ***The authors should highlight these caveats****. It would also strengthen the study if they could obtain sequences from sorted γδ from thymectomy, and from multiple donors, if possible.*

Response:

We are very much in agreement with Reviewer 1 in that we would have liked to have access to more thymoma sample to strengthen this section. Unfortunately, it is not standard clinical practice to bank the thymoma tissue for research purposes following thymectomy. P1 was the only patient who was identified and diagnosed during the duration of our Good's syndrome study and thus we were able plan for the collection of the tumor tissue during the patient's scheduled surgery.

We have updated our discussion to include highlighting the caveats with only a single thymoma analyzed from an FFPE section, (**page 13, line 362–366**):

> "However, the conclusions that can be drawn from these data are limited, as the analysis was performed on a single thymoma sample. Moreover, because RNA was extracted from formalin-fixed paraffin-embedded tissue sections, we were unable to assess the frequency or phenotypic characteristics of γδ T cells within the broader thymoma tissue."

Minor points

5. The observed clonal overlap of 9.6% in patients P9 and P10 is unusually high given the private nature of TRD clonotypes. The authors should comment on the sequences that were observed to be shared. Were these Vδ2 sequences or did they use other TRDV chains? Were these simple rearrangements with few N nucleotide additions? Or are the shared clonotypes the larger expanded clonotypes from one of the donors? If these samples were sequenced on the same chip this could be a sequencing/assignment error rather than true clonal overlap.

Response:

We were similarly surprised by the degree of overlap between the P9 and P10 TRD repertoires at the time of analysis and had carefully considered the possibility of a technical artefact, as suggested by Reviewer 1.

However, these samples were sorted, processed, and sequenced in separate batches using distinct barcodes, making sample cross-contamination or indexing errors unlikely. We therefore do not believe this observation reflects a technical issue.

To increase transparency, we have now explicitly commented on this overlap in the revised manuscript and included a supplementary table detailing the top shared clonotypes between these donors.

(**page 8, line 216–219**):

> "There was an unusually high clonotype overlap between P9 and P10 of 9.6%. These were predominantly TRDV2 clonotypes with shorter CDR3 sequences that are were clonally expanded in the donors (see **Supplementary Table 4** for the top shared clonotype details)."

Supplementary Table 4

CDR3 sequences	TRDV	TRDJ	P9 frequency	P10 frequency
ACDTGGWGISSLTAQLF	TRDV2	TRDJ2	19.7%	18.3%
ACDTGTGIRNGLI	TRDV2	TRDJ1	23.3%	1.3%
ACDKLRETDKLI	TRDV2	TRDJ1	1.2%	5.6%
ACDSLLGDKGLI	TRDV2	TRDJ1	0.5%	1.4%
ACFSVVLSDKLI	TRDV2	TRDJ1	0.4%	1.0%

6. Expression of CD38 and CD16 is reported on total γδ T cells in Figure 2 rather than in specific γδ subsets. Presumably Vδ1 and Vδ2 antibodies were included in these staining panels, so expression of CD38 and CD16 could be delineated on different γδ subsets. In general, it would be interesting to attempt to relate the phenotyping data to the clonotyping data more in these donors.

Response:

Vδ1 and Vδ2 antibodies were not included in the original immunoprofiling assay, but where we had sample available, we have performed additional immune phenotyping with these markers now included and presented the data in **Supplementary Figure 4**:

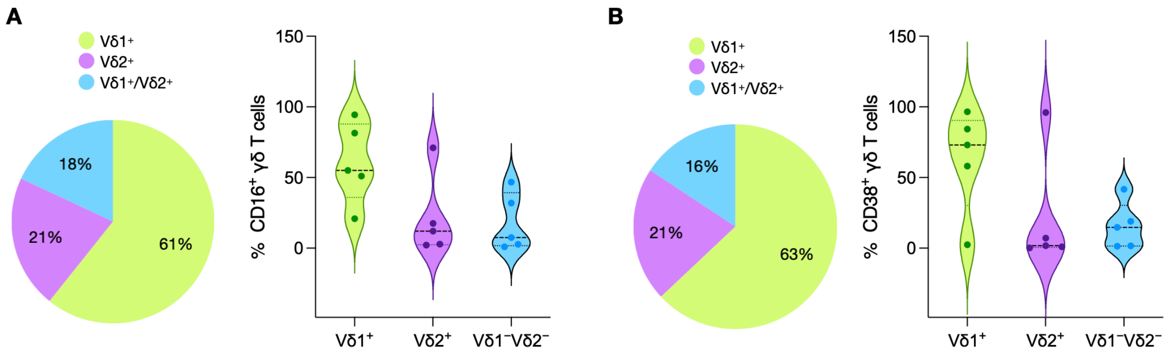

And added the following to the main text (**page 6, line 142-144**):

> "We determined that the observed increase in $CD16^+$ and $CD38^+$ γδ T cells was driven by the $Vδ1^+$ subset (**Supplementary Figure 4**)."

7. In lines 177-179, the authors say TRDJ1 is associated with adult-derived γδ T cells, while TRDJ3 is associated with fetal-derived γδ T cells. While this was shown for Vγ9Vδ2 T cells, to my knowledge this has not been shown to be the case in other Vδ2neg T cell subsets.

Response:

Evidence supporting differential TRDJ usage beyond Vγ9Vδ2 T cells has been reported by Tieppo et al. (2020). In Figure S2 of their study, TRDV1/2/4/5/8 chains predominantly associate with TRDJ1 in adult samples, whereas fetal γδ T cells show a use of TRDJ2 and TRDJ3.

To clarify this point, we have revised the main text to more precisely reflect the published evidence and now explicitly cite this study (**page 7, line 191–192**):

> "We next examined the TCR δ joining (TRDJ) segment usage to assess whether γδ T cells were adult-derived (TRDJ1) or fetal-derived (TRDJ2/3)[1]"

8. In Figure 3E it is surprising there is not much response to anti-CD3/28 beads in the Vδ1 and Vδ1/2neg T cell subsets. Do you have any thoughts as to why this is the case? In other reports I believe Vδ1 cells in PBMC respond ex vivo to anti-CD3/28.

Response:

We agree that the $V\delta1^+$ and $V\delta1^-V\delta2^-$ subsets response to anti-CD3/CD28 stimulation was relatively modest. In our experience, the $V\delta1^+$ γδ T cells exhibit variable responsiveness to TCR-mediated stimulation ex vivo, which may reflect differences in signal 3/cytokine signaling within the stimulation culture. IL-2 was not added to these cultures; the addition of IL-2 is known to augment proliferation and effector cytokine production and may account for the lower magnitude of response observed here compared with some published reports. Notably, $V\delta2^+$ γδ T cells from the same PBMC wells demonstrated robust responses, confirming that the stimulation conditions were effective.

Reviewer #2

We are pleased Reviewer 2 states: ***"This study provides the first analysis of γδ T cells in Good's syndrome… the analysis and cohort is technically sound".***

Please see below for our responses to specific points raised.

Comment 1

The study is very descriptive. While elevated gd T cell frequencies are obvious, it is unclear whether this is due to (i) direct thymic output due to the thymoma, (ii) compensatory proliferation to infections and loss of adaptive immunity (T cells and B cells, (iii) or direct expansion to tumor antigens. Therefore, ***the authors should perform transcriptomic analysis and/or analyze proliferative markers and cell markers for thymic output to better elucidate on potential causes****.*

Response:

We thank reviewer #2 for this suggestion and agree that it would provide clarity regarding the functional state of γδ T cell subsets in Good's syndrome. To address this, we have performed an RNAseq experiment on sorted healthy and patient γδ T cell subsets as well as specifically graphed and discuss the expression of markers of thymic egress.

This new data is presented in **Figure 7**:

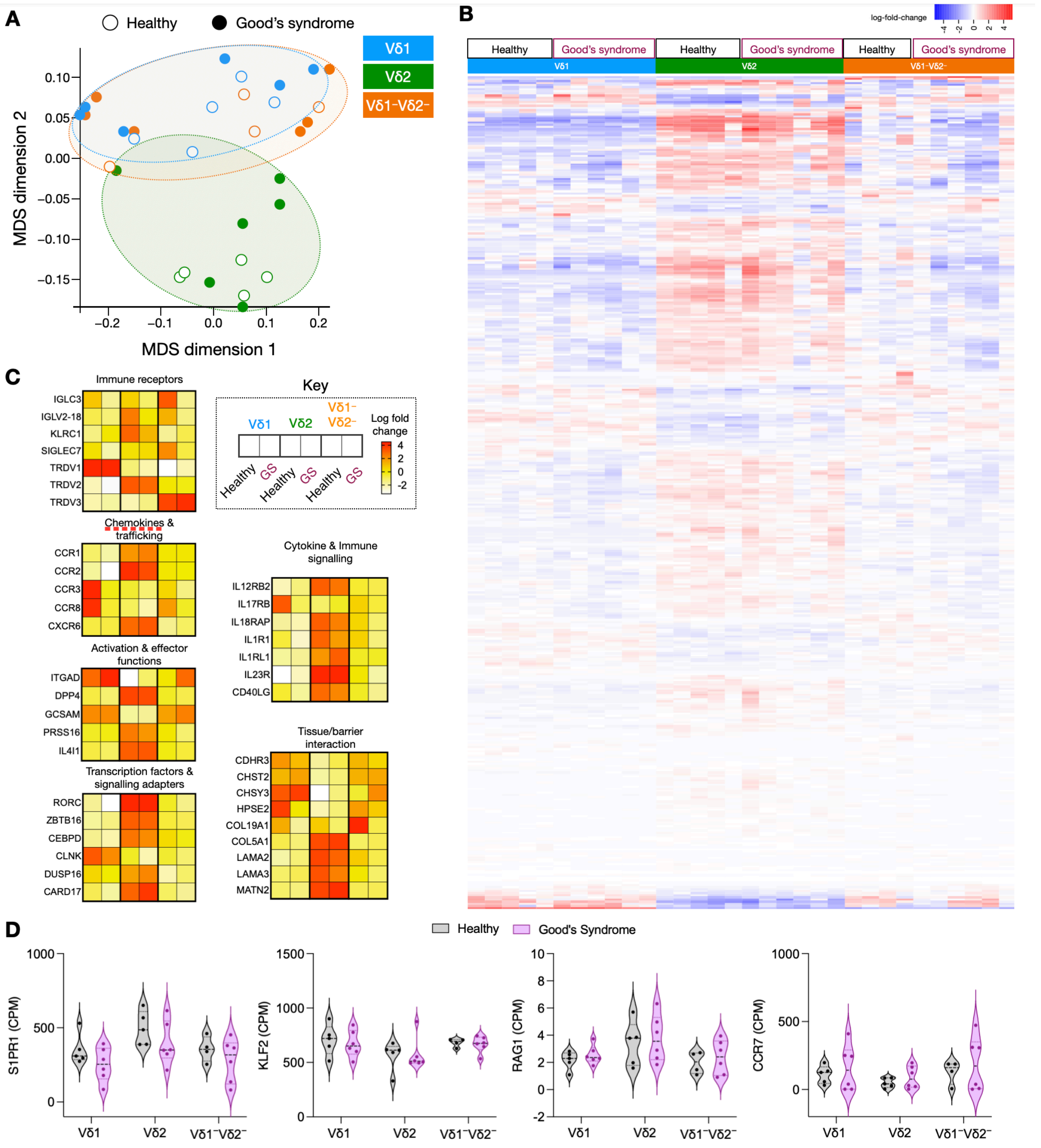

With the following also added to the main text (**page 9-10, line 252-276**):

"To determine whether the γδ T cell subsets in Good's syndrome are functionally distinct and comparable to those observed in healthy individuals, we performed RNA sequencing on *ex vivo* sorted subsets. Consistent with prior reports Vδ2$^+$ and Vδ2$^-$ γδ T cells exhibited clear transcriptional separation, which was largely maintained between healthy individuals and Good's syndrome patients (**Figure 7A–B**). However, Vδ2$^+$ cells from Good's syndrome patients showed increased dispersion towards the Vδ2$^-$ transcriptional space, suggesting partial convergence towards an adaptive-like state (**Figure 7A)**, in line with the expanded Vδ2$^+$Vγ9$^-$ population we observed in certain patients (**Supplementary Figure 3**).

At the gene level, differential expression analysis revealed selective alterations in immune-related pathways. Vδ1$^+$ γδ T cells from Good's syndrome patients displayed reduced expression of *CCR3*, *CCR8*, *IL17RB* and *HPSE2* consistent with altered migratory and effector potential. In Vδ1$^-$Vδ2$^-$ subsets, increased *ITGAD* expression alongside reduced *IGLC3*, *SIGLEC7*, *CCR8* and *COL19A1 expression* suggests shifts in adhesion, immune regulation, and tissue interaction

programs. Collectively, these data indicate that while core subset-defining transcriptional programs are preserved, Good's syndrome is associated with targeted rewiring of immune pathways, suggesting that γδ T cells retain lineage identity but undergo context-dependent functional adaptation.

We next examined the expression of genes associated with thymic egress and recent thymic emigrant status, including *S1PR1*, *KLF2*, *RAG1*, and *CCR7* (**Figure 7C**). No significant differences were observed across γδ T cell subsets or between Good's syndrome patients and healthy donors. No significant differences were observed across γδ T cell subsets or between Good's syndrome patients and healthy donors. These findings suggest that the altered γδ T cell composition is unlikely to be driven by differences in recent thymic egress, and instead reflects prior perturbations associated with thymoma and/or peripheral adaptation following disease onset.

Comment 2

The functional consequence on clinical output and phenotypes on the observed changes in the gd T cell compartment are not addressed in detail. **Can the authors provide more details on the clinical features of the individual patients**. *For instance, not in every patient an expansion of Vd3 T cells is evident.*

Response:

We agree that inclusion of all clinical characteristics and phenotypes will assist in assessing the alterations in the γδ T cell compartment. We have now included the following table that provides extended demographic and clinical information from Figure 1, with details of patients baseline characteristics, tumor, clinical presentation, and CMV status.

See **Supplementary Table 2**:

	P1	P2	P3	P4	P5	P6	P7	P8	P9	P10
BASELINE CHARATERISTICS										
**Year of birth**	1950	1971	1955	1964	1945	1965	1962	1961	1967	ND
**Death**	-	-	2023	2025	-	-	-	-	-	-
**Sex**	Male	Male	Male	Male	Female	Male	Female	Female	Female	Male
**Ethnicity**	Singaporean	Mauritian	Vietnamese	ND	Caucasian	Caucasian	Indian	Caucasian	Caucasian	Caucasian
THYMOMA										
**Diagnosis**	2024	2007	2012	2010	2015	2020	2020	2020	2022	57y
**Tumor type**	B2	A	B2	ND	AB	AB	B2	AB	AB	Thymolipoma
**Tumor stage**	I	I	II	ND	I	II	I	I	I	ND
CLINICAL FEATURES										
**Recurrent infection onset**	N/A	2010	2012	ND	2017	2020	2020	ND	2020	57y
**GS Diagnosis**	2024	2011	2012	2010	2017	2020	2020	2019	2023	57y
**Immuno-modulation**	nil	IVIg SSZ	IVIg SCIg	nil	PRED CsA	IVIg PRED	SCIg HCQ MMF	SCIg	PRED IVIg SCIg	MP/DXM TPE IVIg Rituximab
**Major complications** BE Autoimmunity Infections	Severe COVID19 requiring ICU	Septic arthritis: *Salmonella* and *Mycoplasma* *Pseudomonas* sinusitis Reactive arthritis Chronic rhinosinusitis	Recurrent cutaneous HSV (2016) BE *Pseudomonas* bacteremia Pulmonary nocardia	CMV hepatitis and gastritis	Immune cytopenias chronic rhinosinusitis	Immune cytopenias Lichen planus recurrent cutaneous HSV onychomycosis	Lichen planus MAC pneumonia	Interstitial lung disease Enteropathy with norovirus Oral lichen planus	Severe COVID19 with organizing pneumonia	Viral-type pneumonia myasthenia gravis *S. epidermidis* surgical site infection
CMV STATUS										
**IgG serostatus***	+	N/A	N/A	+	+	N/A	N/A	N/A	N/A	ND
**Viremia**	ND	–	+	+	ND	ND	ND	ND	+	ND

*Only applicable for patients not on Ig replacement therapy
BE, bronchiectasis; CMV, cytomegalovirus; CsA, cyclosporin; DXM, dexamethasone; GS, Good's syndrome; HCQ, Hydroxychloroquine; HSV, herpes simplex virus; ICU, intensive care unit; IVIg, intravenous immunoglobulin replacement; MAC, Mycobacterial Avium Complex; MMF, Mycophenolate mofetil; MP, Methylprednisolone; N/A, not applicable; ND, not determined/reported; PRED, Prednisolone; SCIg, subcutaneous immunoglobulin replacement; SSZ, Sulfasalazine; TPE, Therapeutic Plasma Exchange.

Comment 3

The TCR-seq identified several expanded clones, but is extremely descriptive. ***What is the reactivity of theses clones?*** *Can the authors model thymoma in vitro to assess potential changes in phenotypes and TCR repertoires from healthy blood cells upon exposure to the in vitro thymoma to assess the direct effect of the tumor on gd T cells.*

Response:

We agree that defining the antigen specificity and functional reactivity of the clonally expanded γδ T cell subsets identified in the Good's syndrome patients would be of considerable interest.

However, modelling thymoma *in vitro* presents substantial technical challenges. Thymic epithelial tumors are notoriously difficult to culture[2], and there are currently no widely available commercial human thymoma cell lines. Our cohort comprised four distinct thymoma subtypes (A, B, AB, and thymolipoma), and we had access only to formalin-fixed, paraffin-embedded tissue from a single patient, preventing our ability to perform functional co-culture experiments.

More broadly, identifying γδ TCR ligands remains a major challenge in the field. Only a limited number of ligands have been described to date (e.g., Annexin A2, MICA, EPCR), and γδ TCR recognition is challenging due to the absence of classical restricting molecules, relatively low-affinity interactions, and substantial private reactivity. These factors make systematic antigen identification technically difficult and currently limit our ability to define the precise reactivity of the expanded clones identified in our study.

Nonetheless, thymomas provide a unique context in which aberrant expression of tissue-restricted self-antigens, tumor neoantigens, or stress-induced ligands could plausibly drive reshaping of the γδ T cell compartment.

To better highlight these aspects in our study, we have now expanded the clinical information in **Supplementary Table 2** to include thymoma details, as well as performing analysis of the γδ T cell subsets based on tumor type and stage,

See **Supplementary Figure 7**:

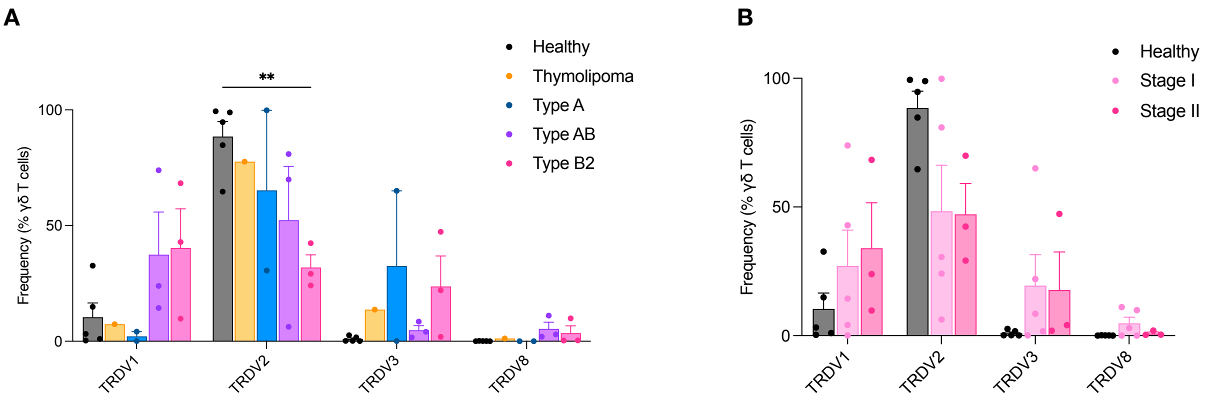

With the following also added to the main text (**page 9, line 232–237**):

> Our Good's syndrome cohort comprised a range of thymoma histological subtypes and stages (**Supplementary Table 2**). We therefore examined whether these clinical variables were associated with alterations in γδ T cell subset distribution. Overall, we observed no significant differences according to tumor stage or histological type. The only exception was Stage B2 thymomas, which demonstrated significantly reduced TRDV2 usage compared with healthy controls (**Supplementary Figure 7).**

For transparency and to facilitate future mechanistic studies, we have also included a table listing the top expanded γδ TCR clonotypes for each patient, in **Supplementary Table 5**:

ID	CDR3 sequences	TRDV	TRDJ	Frequency	Private/ shared
P1	ALPTFLRNPGGYARVDKLI	TRDV1	TRDJ1	1.3%	Private
	ALGEAPHYWGMRGRYTDKLI	TRDV1	TRDJ1	1.1%	Private
	ALGHSQGQWGIGKDTDKLI	TRDV1	TRDJ1	1.0%	Private
P2	ALDKPRWGTQTIKYTDKLI	TRDV3	TRDJ1	29.4%	Private
	ASLQRGILTDKLI	TRDV3	TRDJ1	28.9%	Private
P3	ALTSTGYTDKLI	TRDV3	TRDJ1	11.6%	Private
P4	ACDRRTGGYRDKLI	TRDV2	TRDJ1	21.1%	Shared
	ACDPIFRTGGTQALTAQLF	TRDV2	TRDJ2	11.4%	Shared
	ASHSGYWGINTDKLI	TRDV3	TRDJ1	9.6%	Shared
P5	AYRRTESQFHFRPTTGGQPFRPPYTDKLI	TRDV8	TRDJ1	10.2%	Shared
	ALGEQWPSDILGDTDKLI	TRDV1	TRDJ1	7.8%	Private
	ALGAGYGVSTDKLI	TRDV1	TRDJ1	6.6%	Shared
P6	ACVPDVLPPLHKLI	TRDV2	TRDJ1	47.9%	Shared
P7	ALEDARWLVWWSGIQKYTDKLI	TRDV1	TRDJ1	38.7%	Shared
P8	ACDTVGDKGDKLI	TRDV2	TRDJ1	10.8%	Shared
	ACDTVSPTQLGGYTDKLI	TRDV2	TRDJ1	9.7%	Private
P9	ACDTGTGIRNGLI	TRDV2	TRDJ1	23.3%	Shared
	ACDTGGWGISSLTAQLF	TRDV2	TRDJ2	19.7%	Shared
P10	ACDTGGWGISSLTAQLF	TRDV2	TRDJ2	18.3%	Shared

Shared indicates CDR3 sequence detected in at least one other individual. Private indicates not detected in any other individuals in this cohort. TRDJ, T-cell receptor delta chain joining region; TRDV, T-cell receptor delta chain variable region.

In addition, we have expanded the Discussion to further consider the potential nature of γδ T cell recognition in Good's syndrome thymomas (**page 12, line 317–324**):

> "It is possible that the presence of the thymoma in Good's syndrome reshapes the γδ T-cell subset composition. This may occur through altered thymic output, potentially driven by aberrant expression of autoantigens or disruption of normal thymic selection cues provided by thymic epithelial cells. Alternatively, changes in subset composition could reflect tumor-associated antigen-driven expansion, including responses to dysregulated self-antigens, stress-induced ligands, or tumor neoantigens recognized by γδ TCRs. However, as the antigenic landscape of thymomas in Good's syndrome is poorly understood, it warrants further investigation."

Comment 4

The authors point out that expansion of Vd3 T cell and Vd8 T cells are evident. Given the frequencies among all gd T cells and within individual donors, ***I would rather state that Vd1, Vd3 and Vd8 T cells are elevated in these patients****. Also, I would not use expansion, as it is not clear whether the cells are really expanding or just show a higher abundance due to absence of other immune cells.*

Response:

We thank reviewer 2 for their perspective on this, and agree it is important to distinguish a true expansion from changes in abundance and to also include the Vδ1⁺ subset increase in the Vδ2⁻ compartment. We have altered the use of "expansion" to only discussions of cell number related data or in cases of "clonal expansion" and for other cases we now use "high abundance" or "high frequency".

Comment 5

In Figure 2 can the authors also ***plot gd T cells, Vd2 T cells, and Vd1 T cells among all lymphocytes****, which may show only abundance of Vd1 and Vd3 T cells in Good's syndrome. Also in the related supplement absolute cell counts for gd T cells should be shown.*

Response:

We thank Reviewer 2 for this suggestion, and we have now calculated the total counts for the γδ T cell and the Vδ1⁺/Vδ2⁺/DN subsets to provide clarity around whether these alterations in the compartments are true expansions of specific γδ T cell subsets.

This new data is now presented in **Figure 3B**:

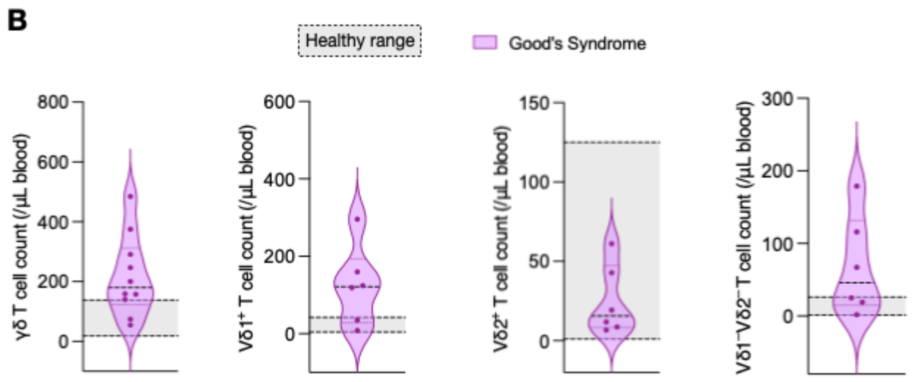

We have also updated the main text to include discussion of the changes in both Vδ1 and Vδ1/2neg subets (**page 6, line 139-142**):

"When γδ T cells were assessed as absolute counts, the majority of Good's syndrome patients exhibited elevated numbers of γδ T cells beyond that of the healthy range (**Figure 3B**). This expansion was driven by increased numbers of both Vδ1⁺ and

$V\delta1^{-}V\delta2^{-}$ subsets, whereas $V\delta2^{+}$ γδ T cell counts remained within the normal healthy range across all patients"

Reviewer #3

We appreciate Reviewer 3 stating: "**The authors use the rare Good's syndrome to study Tgd development in humans. This is an elegant approach to better characterize this otherwise understudied cell population.**"

Please see below for our responses to the specific point raised.

Comment 1

Functional insight is limited. Could the authors apply some additional bioinformatics tools such as Monocle analysis to better understand intrapatient transcriptomic heterogeneity and potentially developmental trajectories.

Response:

We agree with Reviewer 3 that insight into intrapatient transcriptomic heterogeneity would strengthen the functional interpretation of γδ T cells in Good's syndrome. In response to this suggestion, we have generated new transcriptomic data by performing bulk RNA sequencing on sorted DN, Vδ1, and Vδ2 γδ T cell subsets, enabling direct comparison of their transcriptional programs within patients.

This new data is presented in **Figure 7**:

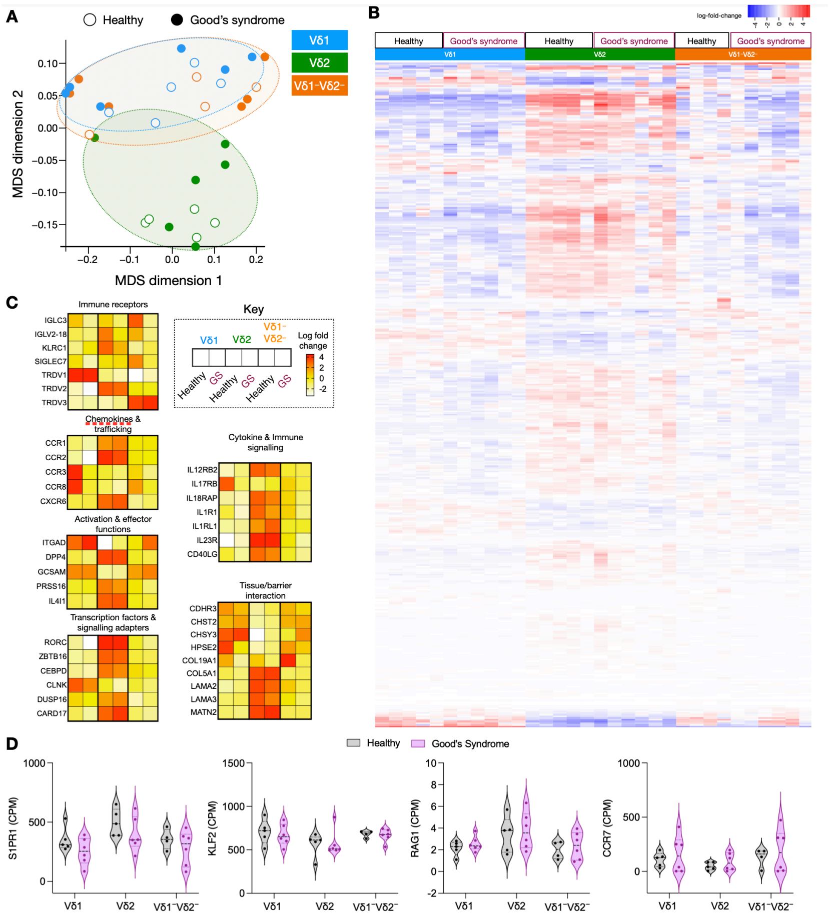

With the following also added to the main text (**page 9–10, line 252–268**):

> "To determine whether the γδ T cell subsets in Good's syndrome are functionally distinct and comparable to those observed in healthy individuals, we performed RNA sequencing on *ex vivo* sorted subsets. Consistent with prior reports $V\delta2^{+}$ and $V\delta2^{-}$ γδ T cells exhibited clear transcriptional separation, which was largely maintained between healthy individuals and Good's syndrome patients (**Figure 7A–B**). However, $V\delta2^{+}$ cells from Good's syndrome patients showed increased dispersion towards the $V\delta2^{-}$ transcriptional space, suggesting partial convergence towards an adaptive-like state (**Figure 7A)**, in line with the expanded $V\delta2^{+}V\gamma9^{-}$ population we observed in certain patients (**Supplementary Figure 3**).
>
> At the gene level, differential expression analysis revealed selective alterations in immune-related pathways. $V\delta1^{+}$ γδ T cells from Good's syndrome patients displayed reduced expression of *CCR3*, *CCR8*, *IL17RB* and *HPSE2* consistent with altered migratory and effector potential. In $V\delta1^{-}V\delta2^{-}$ subsets, increased *ITGAD* expression alongside reduced *IGLC3*, *SIGLEC7*, *CCR8* and *COL19A1 expression* suggests shifts in adhesion, immune regulation, and tissue interaction programs. Collectively, these data indicate that while core subset-defining transcriptional programs are preserved, Good's syndrome is associated with targeted rewiring of immune pathways, suggesting that γδ T cells retain lineage identity but undergo context-dependent functional adaptation.

While trajectory-based approaches such as Monocle are not applicable to bulk RNA-seq data, our analysis nonetheless provides important functional insight by defining subset-specific gene expression signatures and highlighting selective alterations in Good's syndrome patients' immune pathways. These data substantially strengthens our ability to infer functional states of γδ T cells in this disorder.

References

1. Tieppo P, *et al*. The human fetal thymus generates invariant effector gammadelta T cells. *J Exp Med* **217**, (2020).

2. Muller D, *et al*. Cell Culture Models for Translational Research on Thymomas and Thymic Carcinomas: Current Status and Future Perspectives. *Cancers (Basel)* **16**, (2024).

Reviewer 1
Comment 1
While the authors have reassured me that the large clonal overlap between patients 9 and 10 is not a result of a sequencing error and could be due to a relatively simple, common rearrangement, I am concerned by some of the other clonotypes listed as shared between donors in the new Supplementary Table 5. Some of these clonotypes are extremely complex and I can tell they contain many N nucleotide additions (though without the actual CDR3 nucleotide sequence I cannot say exactly how many) and are therefore extremely unlikely to be generated in two individual donors. One example of this is the sequence from donor P5: AYRRTESQFHFRPTTGGQPFRPPYTDKLIF. The 20 amino acid (60 nucleotide) sequence RTESQFHFRPTTGGQPFRPP must be derived from D gene segments and N nucleotide addition. While TCRd often uses two TRDD gene segments, these segments are relatively short and this still leaves many N nucleotides added (for this sequence I estimate at minimum 40-50 N nucleotides). I would encourage the authors to look carefully at whether the two donors who share this sequence were sequenced on the same chip and if there is any chance that the sequence could have been misassigned, or if contamination could have occurred at another point in the sample preparation. I only highlight this because there is a misconception in the field that it is common/likely to identify shared gd TCR sequences between donors (perpetuated by sequencing errors or sample contamination in several published studies). However, the probability of generating a shared sequence with 40 N nucleotides is 1:4^{40} which is 1:1x10^{24} (approximately 27 billion times larger than the total number of cells in the human body), hence this is extremely unlikely/impossible. The sequence from donor P7 also looks complex and likely to contain many N nucleotides, as do several others.

<u>Response:</u>

For the clonotype overlap analysis, we initially applied a moderate read-count threshold of 10 reads per clonotype. However, we recognise that this threshold allows for the potential inclusion of low-frequency sequencing artefacts, which may lead to the misclassification of "shared" clonotypes. As Reviewer #1 has noted, the apparent sharing of highly complex N-addition clonotypes between donors is consistent with this possibility.

To address this, we have increased the threshold to a more stringent cutoff of 50 reads per clonotype and re-analysed clonotype sharing across donors. Using this more conservative approach, six clonotypes previously classified as "shared" have now been redesignated as "private," including those highlighted by Reviewer 1 with highly complex N-additions in donors P5 and P7 (**Supplementary Table 5)**. The remaining five shared clonotypes are all within the TRDV2 family and display the expected short N-addition profiles. We have also clarified the read-count threshold criteria in the footer of **Supplementary Table 5**.

Supplementary Table 5 – **Top expanded clonotypes for each patient.**

ID	CDR3 sequences	TRDV	TRDJ	Frequency	Private/ shared
P1	ALPTFLRNPGGYARVDKLI	TRDV1	TRDJ1	1.3%	Private
	ALGEAPHYWGMRGRYTDKLI	TRDV1	TRDJ1	1.1%	Private
	ALGHSQGQWGIGKDTDKLI	TRDV1	TRDJ1	1.0%	Private
P2	ALDKPRWGTQTIKYTDKLI	TRDV3	TRDJ1	29.4%	Private
	ASLQRGILTDKLI	TRDV3	TRDJ1	28.9%	Private
P3	ALTSTGYTDKLI	TRDV3	TRDJ1	11.6%	Private
P4	ACDRRTGGYRDKLI	TRDV2	TRDJ1	21.1%	Private
	ACDPIFRTGGTQALTAQLF	TRDV2	TRDJ2	11.4%	Private
	ASHSGYWGINTDKLI	TRDV3	TRDJ1	9.6%	Private
P5	AYRRTESQFHFRPTTGGQPFRPPYTDKLI	TRDV8	TRDJ1	10.2%	Private
	ALGEQWPSDILGDTDKLI	TRDV1	TRDJ1	7.8%	Private
	ALGAGYGVSTDKLI	TRDV1	TRDJ1	6.6%	Private
P6	ACVPDVLPPLHKLI	TRDV2	TRDJ1	47.9%	Shared
P7	ALEDARWLVWWSGIQKYTDKLI	TRDV1	TRDJ1	38.7%	Private
P8	ACDTVGDKGDKLI	TRDV2	TRDJ1	10.8%	Shared
	ACDTVSPTQLGGYTDKLI	TRDV2	TRDJ1	9.7%	Private
P9	ACDTGTGIRNGLI	TRDV2	TRDJ1	23.3%	Shared
	ACDTGGWGISSLTAQLF	TRDV2	TRDJ2	19.7%	Shared
P10	ACDTGGWGISSLTAQLF	TRDV2	TRDJ2	18.3%	Shared

Shared indicates CDR3 sequence detected in at least one other individual at a read count of >50 to remove false-positives. Private indicates not detected in any other individuals in this cohort. TRDJ, T-cell receptor delta chain joining region; TRDV, T-cell receptor delta chain variable region.

We are grateful for Reviewer #1's expertise in γδ TCR analysis and for highlighting this important issue. This revision strengthens the overall conclusions of our study, consistent with our original statement (**Page 8, line 205-207 of the main text**): *"The top expanded clonotypes for each donor are a mixture of subsets, consisting of mostly public TRVD2 subsets and private TRDV1 and TRDV3 subsets (**Supplementary Table 5**)."*

Comment 2

The new Supplementary Table 3 including more information about the patients and their CMV status is useful. However, the legend for this table is a bit confusing at present. They need to clarify that "+" means CMV seropositive while "V+" means CMV viremia.

<u>Response</u>

We have also updated the footer of **Supplementary Table 3** with clarification regarding the CMV + vs +V status as requested by Reviewer 1. I have attached an updated Supplementary materials word document (with the discussed changes tracked) as well as a clean PDF.

Supplementary Table 3 – Sample details for TRD repertoire analysis.

ID	Cohort	CMV status	Number of sorted γδ T cells	TRD sequence reads	Distinct CDR3
**HD1**	Healthy	+	69 575	317 743	995
**HD2**	Healthy	+	43 981	746 718	1 063
**HD3**	Healthy	+	108 200	314 472	7 032
**HD4**	Healthy	+	133 000	294 784	914
**HD5**	Healthy	+	208 000	584 659	2 552
**P1**	Good's syndrome	+	*Blood T1:* 469 700	1 048 260	36 907
			Blood T2: 108 000	585 130	20 812
			Thymoma: N/A	293 449	1 683
**P2**	Good's syndrome	ND	51 000	337 647	1 597
**P3**	Good's syndrome	+V	94 600	314 969	2 361
**P4**	Good's syndrome	+V	157 000	1 142 483	2 420
**P5**	Good's syndrome	+	966 000	1 622 335	4 180
**P6**	Good's syndrome	ND	332 300	1 480 150	4 466
**P7**	Good's syndrome	ND	100 600	1 144 855	4 158
**P8**	Good's syndrome	ND	170 048	473 115	2 587
**P9**	Good's syndrome	+V	302 000	1 110 606	7 071
**P10**	Good's syndrome	ND	105 969	296 351	5 566

ND, not determined; TRD, T cell receptor delta +V, positive CMV viral load +, positive CMV IgG serostatus.

Comment 3

The new RNAseq data and analyses bolster the paper and show that different gd T cell subsets broadly maintain their normal phenotypes in Good's syndrome, although there are some interesting differences highlighted. However, these differences have not been validated at the protein level so the significance of these transcriptional changes should not be overstated.

Response

Indeed, this has only been validated at the RNA level, thus we have added language into the main text when describing these results to make clear that we are only referring to changes observed at the transcriptomic level.
